# Synthesis, single crystal (XRD), Hirshfeld surface analysis, computational study (DFT) and molecular docking studies of (E)-4-((2-hydroxy-3,5-diiodobenzylidene)amino)-N-(pyrimidine)-2-yl) benzenesulfonamide

**DOI:** 10.1016/j.heliyon.2021.e07724

**Published:** 2021-08-06

**Authors:** N. Elangovan, S. Sowrirajan

**Affiliations:** a Department of Chemistry, Arignar Anna Government Arts College, Musiri 621211, Bharathidasan University, Tiruchirappalli, Tamilnadu, India; b Department of Chemistry, King Fahd University of Petroleum and Minerals, Dhahran 31261, Saudi Arabia

**Keywords:** Synthesis, Single crystal, DFT, Drug-likeness, Molecular docking, Sulfadiazine

## Abstract

The Schiff base (E)-4-((2-hydroxy-3,5-diiodobenzylidene)amino)-N-(pyrimidine)-2-yl) benzene sulfonamide (DIDA) compound was synthesis with condensation of 3,5-diiodosalicylaldehyde and sulfadiazine. The compound characterized with FTIR, X-ray crystallography and electronic spectra. The titled compound associated with experimental and theoretical method, DFT used for the theoretical method. The IR was calculated from DFT mode with B3LYP/GENSEP basic set. The electronic spectra computed from TD-DFT method with CAM-B3LYP functional, with IEFPCM solvation model and DMSO used as the solvent. Wave function based properties like localized orbital locator, electron localization function and non-covalent interactions have been studied extensively. The ADMET properties of the compound DIDA indicated that the compound has excellent drug likeness properties and PASS studies showed that it has anti-infective properties, which is confirmed by a docking score of -7.4 kcal/mol.

## 1 Introduction

Schiff base are the compounds containing imine group such as –HC=N- by the reaction of amine with a carbonyl compound or ketone [1]. The compounds are used in the development of numerous potential application and coordination chemistry in a various pharmacological and biological fields [2,3,4,5]. The sulfonamides and its groups –SO_2_-NH- are well-known as simplest molecule to sulfa drugs. This simple compound has a high potential in pharmacological and toxicological activities [6]. This potential is accredited to the exchange of different functional groups without variation of the structural (–SO_2_-NH-) feature [7,8]. Sulfur holding compounds have been used as drugs for burn treatment and disease. The sulfonamides are used as antibiotics to treat infection disease as inhibited agents against anti-thyroid, diuretic, hypoglycaemic, fumer cells and many more other activities [9]. So it looks to continue investigation in this area. Antimicrobial causes engage of numerous artificial organic compounds proficient of preventing the go forward of bacteria with the intention of occupy Para-amino benzoic acid (PABA) exist mostly related to sulfonamides. Sulfonamides attitude the derived of sulfonic acids, sulfonamides are chemically pretty persistent, and they are pathetic acids related to carboxylic acid and amides [10,11,12,13]. The synthesis of novel compound of sulfonamides derivatives and investigate of their biological and chemical behaviors has turn into support not long for biological, drug purpose and pharmaceutical [14]. Molecular docking is most important techniques for predict the biological activity of synthesized compounds and another one important is predict which amino acid or DNA interacted to synthesized compound.

The specific goal of this is confirm the structure of synthesized compound using XRD and predict the biological activity using molecular docking. Hence we are aim to synthesis a series of Schiff base ligand derived from sulfadiazine derivative. In the present study, we are going to synthesis of new Schiff base ligand attained by the condensation of 3,5-diiodosalicylaldehyde by sulfadiazine and characterized by the structure by UV-Vis, XRD and FT-IR. The crystal building of the compound DIDA were characterized using DFT/B3LYP/GENSEP basic set.

## 2 Materials and methods

### 2.1 Materials and instrumentation

Sulfadiazine and 3,5-diiodosalicylaldehyde were purchased from Sigma-Aldrich Company. The solvents DMSO and methanol were purchased from Ponmani & Co (Tiruchirappalli). All the chemical and solvents are AR grade and used for without further purification. The FTIR were recorded from FTIR spectrophotometer using KBr pellets with range of 4000–400 nm. The UV-Vis recorded from Cary UV spectrophotometer with solvent used for DMSO. The X-Ray crystallography (single crystal) was recorded from Bruker, 2016 at Indian Institute of Technology Madras (Chennai). The Hirsfeld surface analysis study formed using the software crystal explorer in version-17.5. The LOL, ELF and RDG studies were carried out from Multiwfn software package. The ADME properties calculated from Swiss ADME online tools.

### 2.2 Synthesis and crystallization

**Figure 1:**
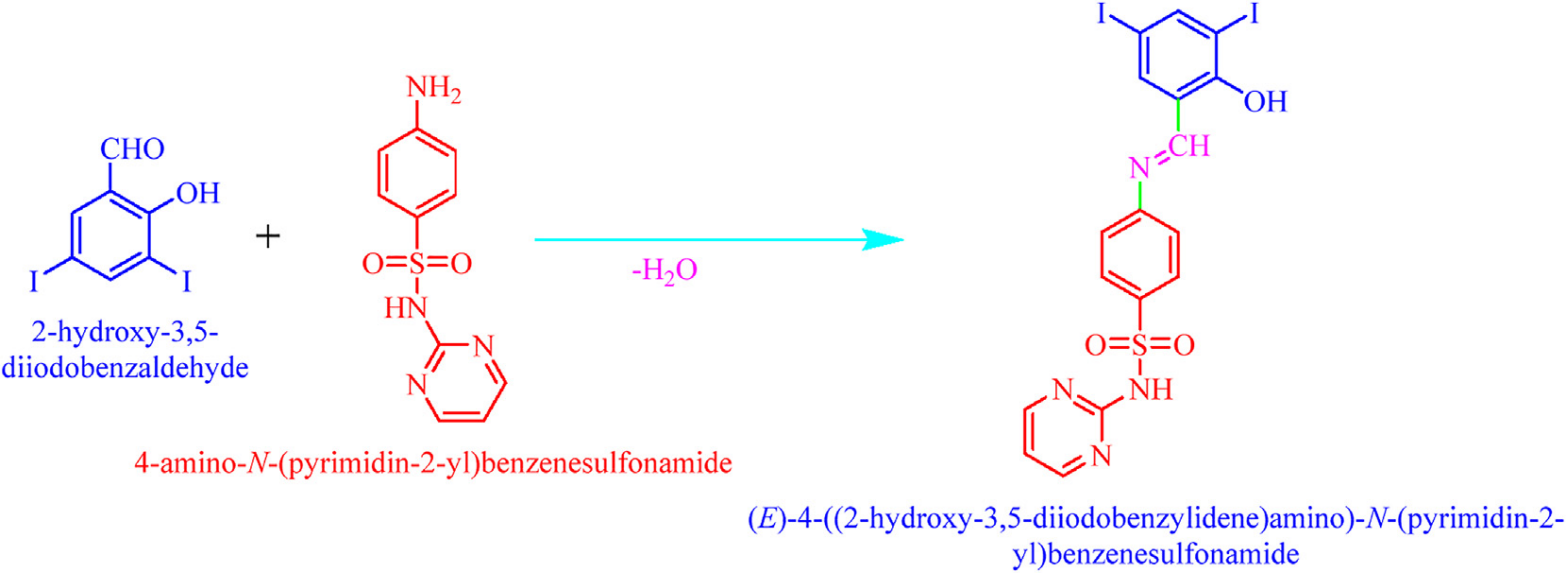
Synthesis of (E)-4-((2-hydroxy-3,5-diiodobenzylidene)amino)-N-(pyrimidine)-2-yl) benzene sulfonamide (DIDA).

A mixture of sulfadiazine (2.50g, 0.01mm) and 3,5-diiodosalicylaldehyde (3.73g, 0.01mm) and grained with a pestle to 3–5 minutes, to this reaction mixture methanol 20mL was added and grained for 5 minutes. The mixture transferred to 100ml RB flask and refluxed for 5–6 hours on completion of reaction as Red-colored solid (E)-4-((2-hydroxy-3,5-diiodobenzylidene)amino)-N-(pyrimidine)-2-yl) benzene sulfonamide (DIDA, Figure 1) was separated out. The obtained solid was recrystallized from DMSO the crystal formed with bottom of the beaker after 20 days slow evaporation [15].

### 2.3 Computational methods

The compound DIDA compute using software Gaussian09 software package, the IR were computed from DFT mode with B3LYP/GENSEP basic set level [15,16]. The vibrational assignments were calculated from VEDA.4 program package [17]. The electronic spectra were calculated from TD-DFT technique with DFT/B3LYP/GENSEP basic set level, with IEFPCM solvation model and DMSO as the solvent [18,19,20]. The NBO, NPA and optimized geometry was calculated from same basic set level. The MD analyses were considered from Autodock/Vina program, result visualization and protein modification using software Discovery studio visualizer. Wave functions were calculated from multiwfn software.

## 3 Results and discussions

### 3.1 Structural geometry analysis

**Figure 2:**
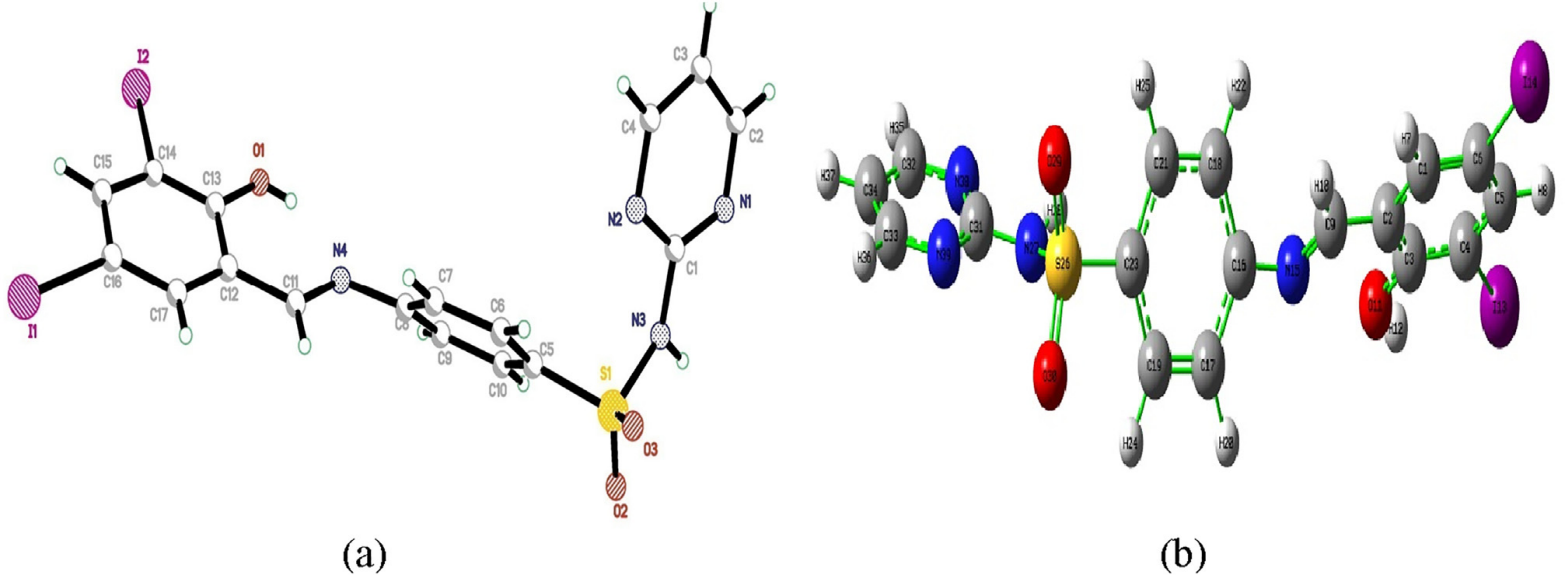
(a) ORTEP (b) optimized structure of the (E)-4-((2-hydroxy-3,5-diiodobenzylidene)amino)-N-(pyrimidine)-2-yl) benzene sulfonamide (DIDA).

The red color single crystal collected from slow evaporation in DMSO solution medium and the dimensions is 0.460 × 0.130 × 0.050 mm3. The molecular geometry of the studied compound is showed for Figure 2. Detailed data collection, crystal data and refinement are shown in Table 1. The intensity were collected from 296K (temperature) at stone image plate diffraction system using MoKα graphite mono-chromate radiation. The structure is solved from direct method using SHELXS-97 program [21]. The refinement and other calculations were carried out from same program. The compound DIDA registered at Cambridge Structural Database (CSD) and registered CCDC number is 2096290.

**Table 1:** Crystallographic data of the compound (E)-4-((2-hydroxy-3,5-diiodobenzylidene)amino)-N-(pyrimidine)-2-yl) benzene sulfonamide (DIDA).

Crystal Data	Data Collection
C17H12I2N4O3S	SADABS (Bruker, 2016)
Mr = 606.17	Tmin = 0.732, Tmax = 0.851
Monoclinic, P21/c	36580 measured reflections
Hall symbol: -P 2ybc	5312 independent reflections
a = 23.3785 (16) Å	3465 reflections with I > 2σ(I)
b = 7.4578 (5) Å	Rint = 0.043
c = 11.3765 (7) Å	θmax = 29.2°, θmin = 0.9°
β = 97.235 (2)°	h = −32→32
V = 1967.7 (2) Å3	k = −10→10
Z = 4	l = −15→15
F (000) = 1152	**Refinement**
Dx = 2.046 Mg m−3	Least-squares matrix: full
Mo Kα radiation, λ = 0.71073 Å	R [F2 > 2σ(F2)] = 0.063
Cell parameters from 9012 reflections	wR (F2) = 0.232
θ = 2.6–23.9°	S = 1.15
μ = 3.33 mm−1	5312 reflections
T = 296 K	252 parameters
Block, yellow	(Δ/σ)max = 0.742
0.10 × 0.10 × 0.05 mm	Δρmax = 1.65 e Å−3, Δρmin = −1.11 e Å−3

The asymmetric unit (Figure 1) contains one 4-((2-hydroxy-3,5-diiodobenzylidene)amino)-N– (pyrimidin-2- yl)benzenesulfonamide. In (I) the parts of 3,5-diiodosalicylialdehyde A (C11–C17/O1/I1/I2), aninilinic group B (C5– C10/N4) and 2-aminopyridine C (C1–C4/N1–N3) are fundamentally planar, with a concentrated deviation of 0.017 (9), 0.039 (8) and 0.039 (8) Å, respectively. The dihedral angle between A/B, A/C and B/C is 46.0 (4)°, 66.6 (4)° and 75.2 (4)°, respectively. The sulfonyl group D (O2/S1/O3) is of course planar. The dihedral angle between A/D, B/D and C/D is 66.3 (6)°, 56.7 (6)° and 70.3 (6)°, respectively. The bond lengths (Allen *et al.*, 1987) and angles are regular.

Optimized structure of the compound DIDA was optimized from DFT mode with B3LYP/GENSEP basic set [15,22,23,24]. The detailed experimental and calculated bond length, bond angle and dihedral angles were listed for table S1. Some main geometrical parameters are discussed given below. The bond is C1–C6, C2–C3, C2–C9, C3–O11, C4–I13, C5–C6, C6–I14, C9–H10, C9–N15, C16–C17, C19–C23, C21–H25, C23–26, S26–O29, N7–C31 with experimental bond length is 1.38, 1.40, 1.48, 1.36, 2.09, 1.39, 2.09, 0.93, 1.25, 1.40, 1.36, 0.93, 1.75, 1.42, 1.37 and related calculated bond length is 1.38, 1.41, 1.46, 1.34, 2.14, 2.13, 1.09, 1.27, 1.40, 1.39, 1.08, 1.79, 1.45, 1.39 respectively. The bond C2–C1–C6, C6–C1–H7, C1–C2–C9, C3–C4–C5, C5–C4–I13, H10–C9–N15, C19–C17–H20, C17–C19–H24, C19–C23–S26, C23–S236–O29, N38–C31–N39, C31–N38–C32 with experimental bond angle is 119.5, 120.3, 117.8, 122.1, 118.5, 119.4, 120.2, 120.1, 120.0, 109.2, 126.6, 115.1 and related calculated bond angle is 121.4, 120.1, 116.2, 122.4, 118.4, 120.7, 120.9, 120.9, 119.3, 108.4, 127.3, 115.4 respectively, finally the bond C6–C1–C2–C9, C2–C1–C6–C5, C1–C2–C9–N15, C2–C3–C4–I13, O11–C3–C4–C5, I13–C4–C5–C6, C4–C5–C6–I14, C2–C9–N15–C16, N15–C16–C18–C21, C17–C19–C23–S26, C18–C21–C23–S26 with experimental dihedral angle is 177.8, -4.1, -176.5, -179.4, -178.7, 178.4, -178.5, -172.8, 177.6, -174.6, 176.1 and related calculated dihedral angle is 179.9, -0.01, -176.8, 179.9, 179.7, 179.9, -179.8, -177.3, 178.7, -179.5, 178.5 respectively, the experimental and calculated value is good agreement.

**Figure 3:**
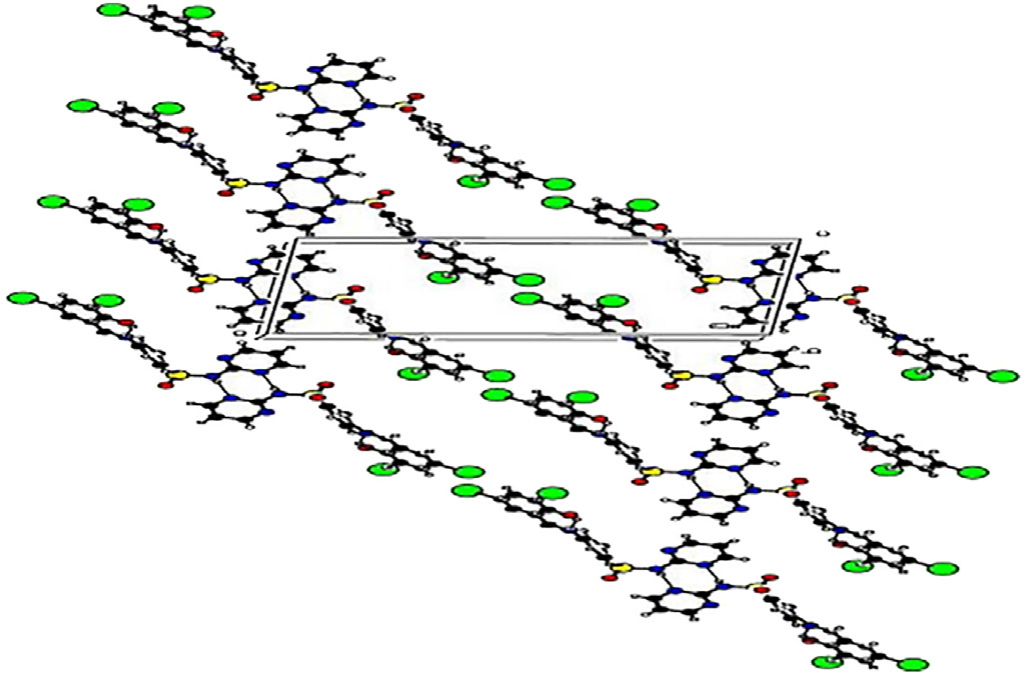
Crystal packing diagram of (E)-4-((2-hydroxy-3,5-diiodobenzylidene)amino)-N-(pyrimidine)-2-yl) benzene sulfonamide.

In this crystal packing (Figure 3), the inversion-centre-related 2-aminopyrimidine are also base-paired *via* N–H···N hydrogen bonds involving the pyrimidine N atom and the 2-amino group. This type of base pairing, also with an *R*2 2 (8) (Bernstein *et al.*, 1995) ring motif, has been observed in many diaminopyrimidinecarboxylate salts (Stanley *et al.*, 2005). In addition, there is a typical intramolecular O–H···N hydrogen bond exists between the hydroxy –OH group and the aninilinic –N group, to form a six-membered hydrogen-bonded ring. The molecules are interlinked in the form of two-dimensional network parallel to the *bc* plane.

O– and N-bound hydrogen atoms were located in a difference Fourier maps and refined freely [O1–H1O1 = 0.69 (12) Å and N3–H1N3 = 0.69 (12) Å]. The remaining hydrogen atoms were positioned geometrically (C–H = 0.93 Å) and were refined using a riding model, with *U*iso(H) = 1.2 *U*eq(C).

### 3.2 Hirshfeld surface analysis

**Figure 4:**
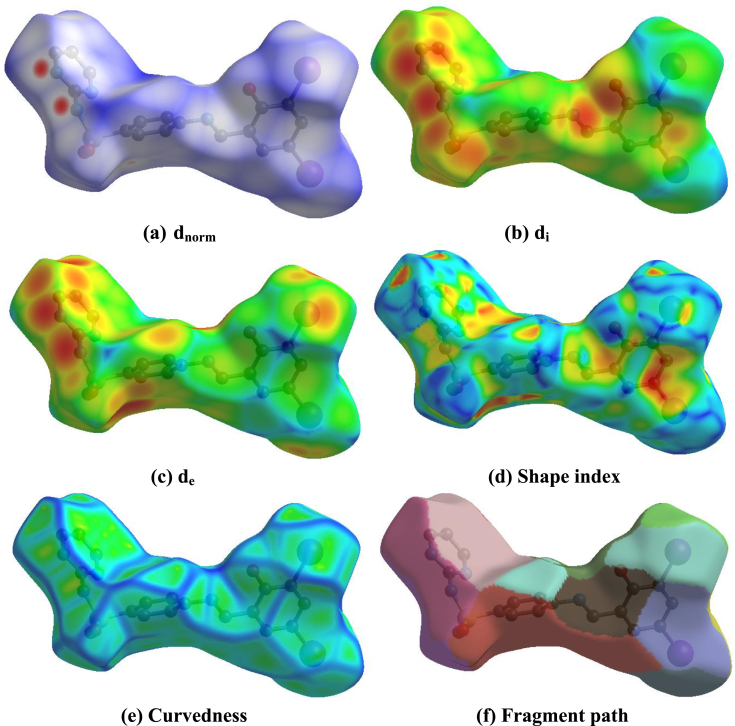
Hirshfeld surface map (a) d_norm_, (b) d_i_, (c) d_e_, (d) Shape index, (e) Curvedness and (f) Fragment path of the compound (E)-4-((2-hydroxy-3,5-diiodobenzylidene)amino)-N-(pyrimidine)-2-yl) benzene sulfonamide (DIDA).

The Hirshfeld surface analysis surrounding the molecule is defined by the points where the contribution to electron density from the molecule of concern is equal to the contribution from other molecule. The isosurface two distances are defined which d_e_ and d_i,_ the d_e_ is distance from the nearest nucleus in external to the surface and d_i_ is the distance from the nearest nucleus in internal to the surface. The normalized contact distance (d_norm_) based on both d_e_, d_i_ and Vander Walls radii of the atom. The d_norm_ value is negative the intermolecular contact are shorter than the Vander Walls separations and d_norm_ value is positive the intermolecular contacts is longer than the Vander Walls separations. The Hirshfeld surface map (d_norm_) using white-blue-red color scheme, while the red is higher light and shorter contacts; the blue is longer contacts and white is contacts around the Vander Walls separations. The d_norm_, d_e_, d_i_, curvedness and shape index value ranges is -0.1352 to 1.5237, 1.4449 to 3.1618, 1.4441 to 3.1673, -4.0000 to 0.4000 and -1.0000 to 1.0000 respectively. The d_e_, d_i_, d_norm_, curvedness and shape index surface map are shown in Figure 4. The d_e_ represents the distance of nearest any surface point to the interior atom and d_i_ represents the distance of nearest surface to the exterior atom and Vander Walls ration of the atom.

**Figure 5:**
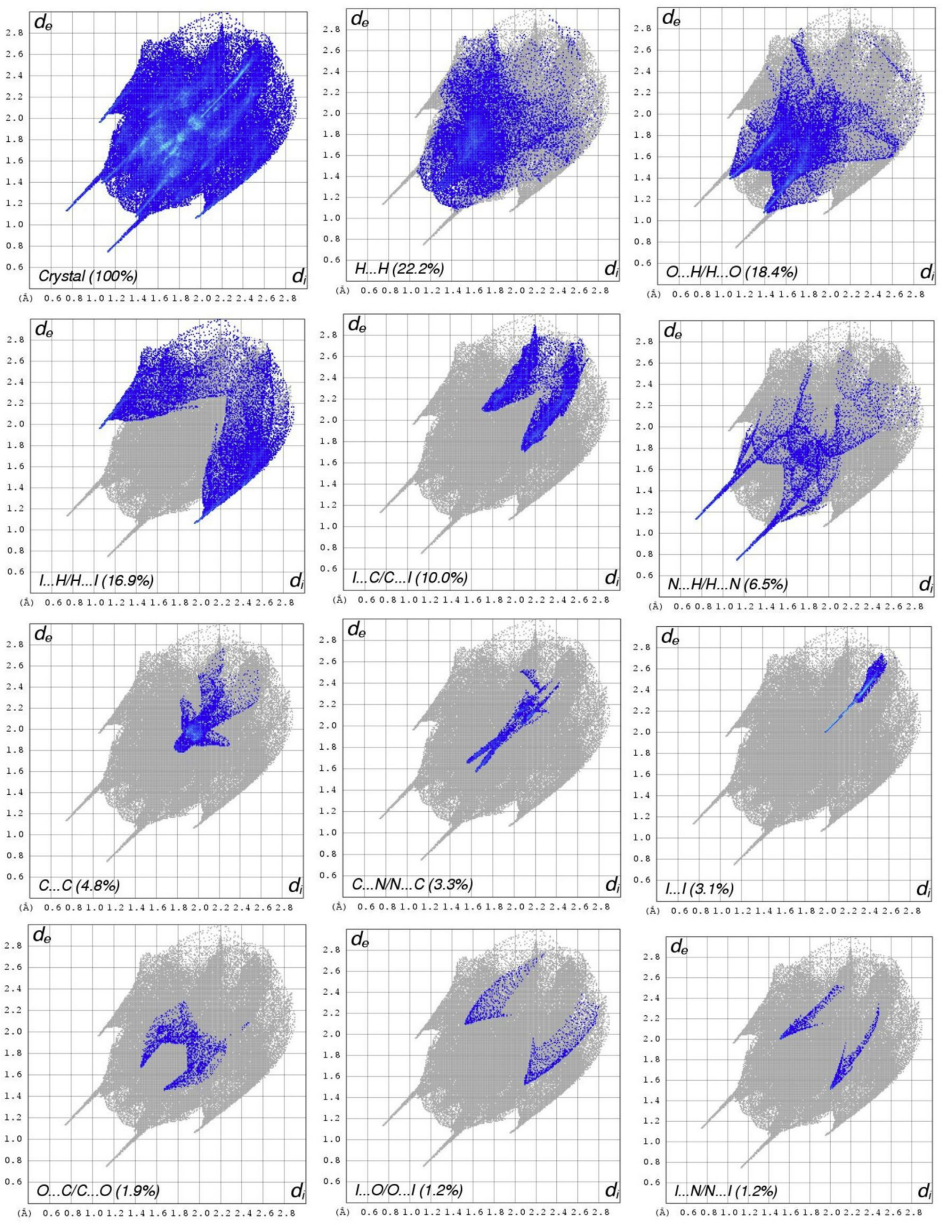
Two dimensional fingerprint plot of the compound (E)-4-((2-hydroxy-3,5-diiodobenzylidene) amino)-N-(pyrimidine)-2-yl) benzene sulfonamide (DIDA).

Visualize and analyze of the inter-molecular interactions is very important tools for three dimensional surface. The three dimensional surface of DIDA are shown in Figure 4. The red color characterize negative and near contacts of three dimensional values of the surface corresponding to the N–H···N and O–H···N interactions [21]. The two dimensional fingerprint plot analysis are shown in Figure 5, which is inter-molecular associates and calculation distribution on the Hirshfeld surface analysis. The percentage of contacts and donate to the total Hirshfeld surface are given below; H···H (22.2%), O···H/H···O (18.4%), I···H/H···I (16.9), I···C/C···I (10.0%) C···H/H···C (9.7%), N···H/H···N (6.5%), C···C (4.8%), C···N/N···C (3.3%), I···I (3.1%), O···C/C···C (1.9%), O···I/I···O (10.2%), I···N/N···I (1.2%) and others (0.8%) are shown in Figure 5.

### 3.3 Vibrational spectral analysis

**Figure 6:**
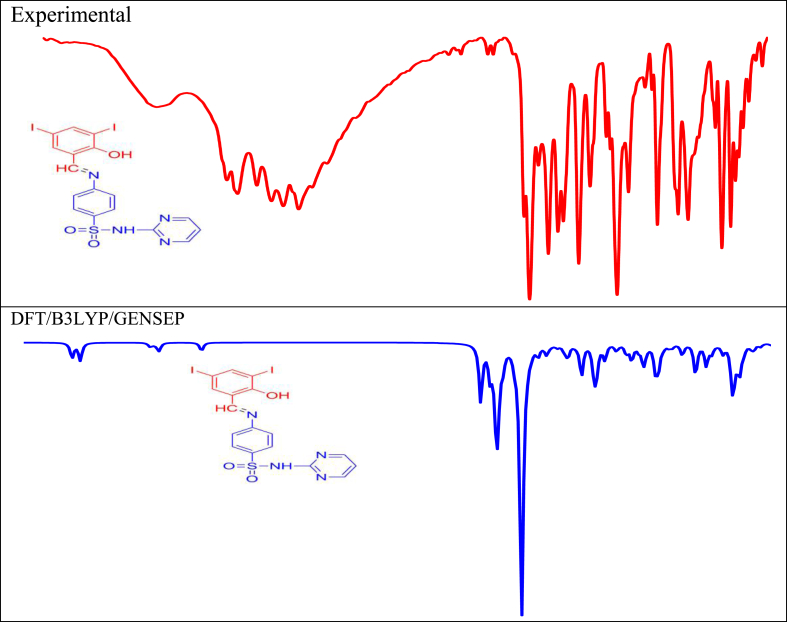
Experimental and calculated FTIR spectra of (E)-4-((2-hydroxy-3,5-diiodobenzylidene) amino)-N-(pyrimidine)-2-yl) benzene sulfonamide (DIDA).

The newly synthesized compound DIDA contains 39 atoms and 111 modes of vibrations and presented in C1 point group, and contains 38 stretching vibrations, 37 bending vibrations, 36 torsion vibrations and 30 CH vibrations are presented with ascending cause is 0.9651 cm–1 [15,25,26,27,28]. The potential energy distribution analysis (PED) were computed from VEDA.4 program and presented in Figure 6, and full assignment is showed at table S4. The important assignment is discussed below.

#### 3.3.1 NH vibrations

Generally 3400-3300 cm-1 is presented at NH vibrations, in this part the NH vibration were presented at 3497 cm-1 experimentally observed [15]. The calculated stretching frequencies are presented at 3506 cm-1, with PED assignment is 100%, corresponding to νNH. The replicated bending vibrations are presented to 1618, 1429, 1426, 1404, 1385, 1353, 1300 and 1185 cm-1 with PED consignment is 11%, 11%, 15%, 11%, 25%, 40%, 37% and 16% due to βCNH respectively, the scaled torsion vibration are presented at 993, 987, 982, 965, 794 and 526 cm-1 with PED influence is 60%, 73%, 21%, 81%, 18% and 12% due to τCCNH respectively. They are closer for calculated and experimental values.

#### 3.3.2 OH vibrations

The OH stretching vibration is presented at 3433 cm-1 in experimental part. The simulated widening shaking are presented at 3469 cm-1, with PED involvement is 100% due to νOH, and ascended bending vibrations is 1314, 1264, 1215, 1085 with PED contribution is 15%, 18%, 13%, 22% corresponding to βCOH, respectively. The virtual torsion vibration is presented at 429 and 422 cm-1, and PED assignment is 19% and 73% due to τCCOH, respectively.

#### 3.3.3 CH vibrations

The CH stretching vibrations experimentally detected at 3084 and 2936 cm-1. The scaled vibrations noted at 3146, 3133, 3132, 3120, 3112, 3108, 3104, 3102, 3101 and 2908 cm-1, and PED contribution is 85%, 99%, 99%, 93%, 75%, 80%, 75%, 99%, 94% and 100% corresponding to νCH respectively, and scaled simulated bending vibrations noted at 1574, 1556, 1426, 1404, 1385, 1352, 1314, 1287, 1236, 1215, 1185, 1174, 1152, 1105, 1095, 1085, 1072 and 1044 cm-1, with PED involvement is 16%, 10%, 17%, 30%, 36%, 26%, 12%, 84%, 19%, 15%, 14%, 55%, 15%, 54%, 12%, 10%, 41%, 11% due to βCCH respectively. The simulated scaled torsion vibrations observed at 990, 973, 908, 888, 856, 843 and 562 cm-1, with PED assignment is 62%, 79%, 62%, 62%, 71%, 77% and 13% due to τCCCH respectively.

#### 3.3.4 CN vibrations

In experimental CN stretching vibration noted at 1610 cm-1. The scaled extending vibration is 1618, 1544, 1515, 1429, 1353, 1227, 1185, 1152 and 866 cm-1, and PED involvement is 65%, 49%, 29%, 43%, 18%, 68%, 46%, 19% and 66% corresponding to νCN respectively. The simulated bending and torsion vibrations noted at 1544, 1515, 043, 946 and 807, 794, 562, 550, 526, 499, 131, 90, 75, 38, 21, 12, 8 cm-1, and PED assignment is 11%, 11%, 46%, 47% and 13%, 48%, 13%, 40%, 18%, 14%, 48%, 10%, 32%, 16%, 10%, 48%, 78% due to βCCN and τCCCN respectively.

### 3.4 Frontier molecular orbital analysis

**Figure 7:**
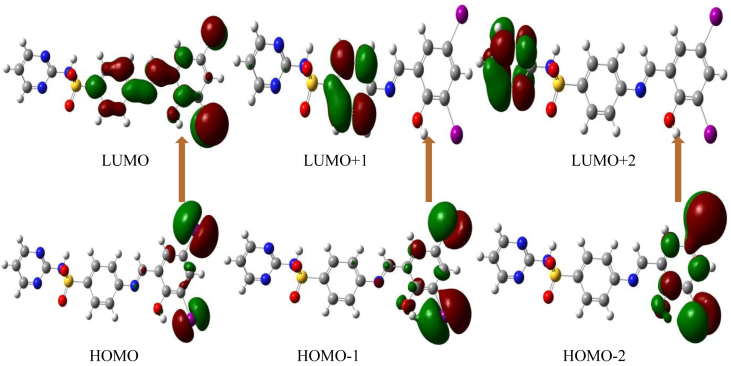
HOMO-LUMO energy diagram of the compound (E)-4-((2-hydroxy-3,5-diiodobenzylidene) amino)-N-(pyrimidine)-2-yl) benzene sulfonamide (DIDA).

Frontier molecular orbital analyses are most important role in electrical and chemical response on the synthesized compound [15,29]. The HOMO is electron donor and LUMO is electron acceptor [30]. Titled compound DIDA HOMO-LUMO energy shown in Figure 7, in this synthesized compound the HOMO and LUMO energy is -4.5 eV and -4.4 eV, with energy gap is 0.1 eV. Generally higher the HOMO-LUMO energy gap the titled compound is hardness and lower the HOMO-LUMO energy the titled compound is softness. But HOMO-I to LUMO+1 the energy gap is 1.0 eV and HOMO-2 to LUMO+2 energy gap is 1.3 eV, conclusion of this studies increase the LUMO the energy gap is increased. HOMO-LUMO global softness is very high which is 20.0, the chemical potential is almost same which is -4.45, -4.1 and -4.05; increase the HOMO-LUMO energy gap, decrease the chemical potential electron accepting power, electronegativity and electron donation power shown in Table 2. Molecular orbital is play important role in the understanding the chemical reactivity in various chemical reactions. The electrophilicity index can be represented to measure of energy lowering due to maximal electron flow between acceptor and donor.

**Table 2:** Frontier Molecular Orbital properties of (E)-4-((2-hydroxy-3,5-diiodobenzylidene) amino)-N-(pyrimidine)-2-yl) benzene sulfonamide.

Property	HOMO-LUMO	HOMO-1-LUMO+1	HOMO-2-LUMO+2
εHOMO	-4.5	-4.6	-4.7
εLUMO	-4.4	-3.6	-3.4
Energy gap ΔE	0.1	1	1.3
Ionisation energy (*I* = εHOMO = -HOMO)	4.5	4.6	4.7
Electron Affinity (*A* = εLUMO = -LUMO)	4.4	3.6	3.4
Global hardness (*η* = (*I-A*)/2)	0.05	0.5	0.65
Global softness (*S =1/η)*	20	2	1.54
Chemical Potential (μ = -(*I* + *A*)/2)	-4.45	-4.1	-4.05
Electronegativity (χ = -μ)	4.45	4.1	4.05
Electrophilicity index (ω = μ2/2*η*)	0.70	2.05	2.31
Nucleophilicity index (*N* = 1/ω)	1.42	0.49	0.43
Electronaccepting powsr ω+ = *A*2/2(*I-A*)	22	1.8	1.31
Electrondonating power ω+ = *I*2/2(*I-A*)	22.5	2.3	1.81

The HOMO is located at iodine atoms and LUMO is located at iodine atoms and phenyl ring [31,32,33]. The most important parameters like ionization energy, hardness and softness, electronegativity, electron accepting power, electron donating power and electron affinity are listed in Table 2.

### 3.5 Molecular electrostatic potential

**Figure 8:**
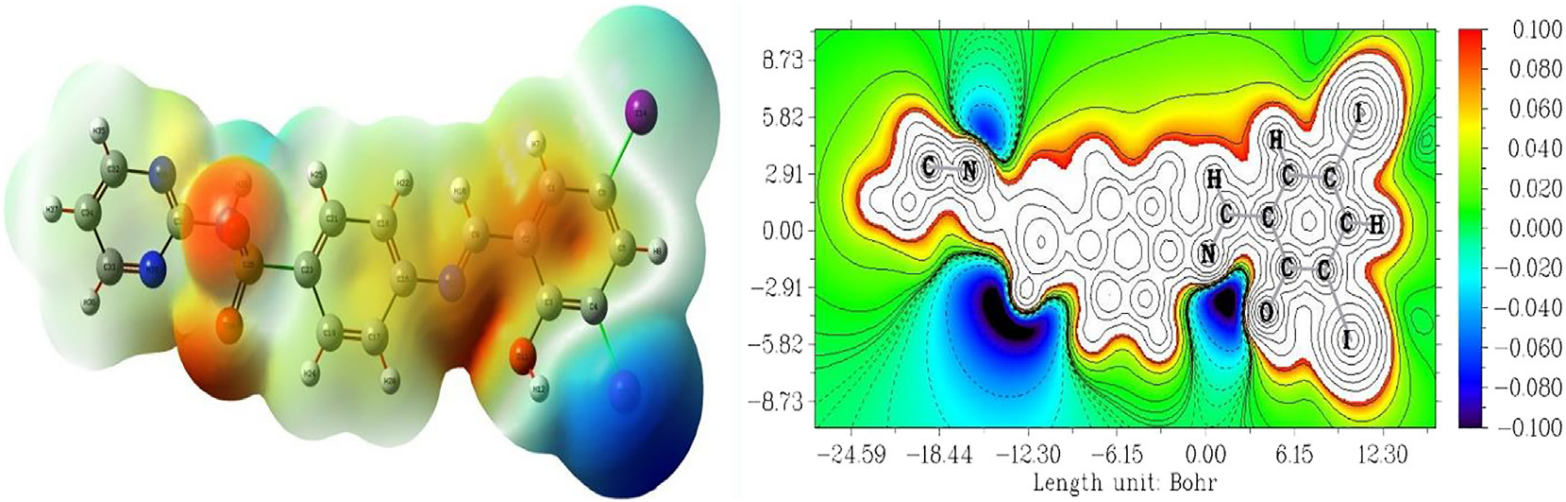
Molecular electrostatic potential (a) color filled surface map (b) counter line map of (E)-4-((2-hydroxy-3,5-diiodobenzylidene) amino)-N-(pyrimidine)-2-yl) benzene sulfonamide.

Molecular electrostatic potential is determined by DFT method with B3LYP/GENSEP basic set method, the MEP predicts the nucleophile and electrophile attack of the molecule [15,16,34]. The more electron rich region showed red color and the more electron poor region showed blue color [18]. The electron rich and poor region is shown in Figure 8. The negative potential region is located at electronegative atoms like O and N and positive regions are located at over the iodine atom. Sulfur atom has less electronegative potential compare to other atoms. The positive and negative electrostatic potential is indicated to nucleophile and electrophile attraction [19]. Electronic spectra shows four absorption peaks like 260, 268, 319 and 364 in experimental section, this is can be due to π- π∗ and n- π∗ transitions.

### 3.6 Electronic properties

**Figure 9:**
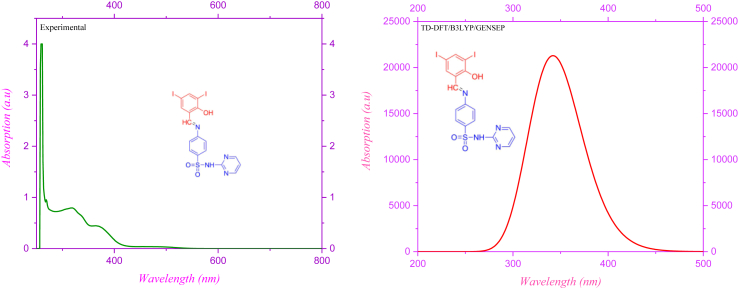
Electronic spectra of (E)-4-((2-hydroxy-3,5-diiodobenzylidene) amino)-N-(pyrimidine) -2-yl) benzene sulfonamide (DIDA).

Electronic spectra show good light harvesting efficiency in the compound DIDA [15,35]. In this part compared to experimental and theoretical electronic spectra [36]. Both of the electronic spectra's were taken from DMSO solvent medium [37]. Theoretical method used at TD-DFT method with B3LYP/GENSEP basic set, and IEFPCM solvation model [38,39]. The TD-DFT mode shows two peaks, but only one 346 nm is significant and oscillator strength is 0.41. Experimental electronic spectra shows four absorption peaks which is 260 268, 319 and 364 nm, and oscillator strength is 4.00, 0.95, 0.79 and 0.45. The first and second peaks dismiss due to π- π∗ transitions, and third and fourth is n- π∗ transitions, the comparison electronic spectra are shown in Figure 9.

### 3.7 Mulliken atomic charge

The Mulliken atomic charges of the compound were considered by DFT way with B3LYP/GENSEP basic set [15,34]. The Mulliken atomic charge of the atom is presented in Table 3. In the compound DIDA 8 carbon atoms, 12 hydrogen atoms, one sulfur atom and two iodine atoms are strongest positive region. 3 oxygen atoms, 4 nitrogen atoms and 9 carbon atoms are strongest negative region. In this structure carbon atom both negative and positive charges, S26 (1.09) highest positive charge due to the highly influence of negative atoms oxygen, and C6 (-0.55) highest negative charge due to the influence of surrounding atoms, H7, H8, H22, H24 and H37 are nearest same positive charges [18,19].

**Table 3:** Mulliken atomic charges of (E)-4-((2-hydroxy-3,5-diiodobenzylidene) amino)-N-(pyrimidine)-2-yl) benzene sulfonamide (DIDA).

Atom	Charge	Atom	Charge	Atom	Charge	Atom	Charge
1 C	0.075188	11 O	-0.380408	21 C	-0.031393	31 C	0.370467
2 C	-0.120872	12 H	0.254488	22 H	0.141164	32 C	0.087022
3 C	0.385645	13 I	0.254113	23 C	-0.329356	33 C	0.119025
4 C	-0.616496	14 I	0.226283	24 H	0.144205	34 C	-0.20479
5 C	0.171439	15 N	-0.337708	25 H	0.150892	35 H	0.158852
6 C	-0.549406	16 C	0.069826	26 S	1.093954	36 H	0.161421
7 H	0.137861	17 C	-0.048346	27 N	-0.532906	37 H	0.142253
8 H	0.136333	18 C	-0.064901	28 H	0.292856	38 N	-0.321262
9 C	0.145294	19 C	-0.043965	29 O	-0.526619	39 N	-0.332379
10 H	0.120958	20 H	0.135706	30 O	-0.534438		

### 3.8 Natural population analysis

The NPA revels that the distributions of electron in various sub shell [15,40]. The addition of electrons in the valance, core and Rydberg sub shell were listed in Table 4 [41,42,43]. The values of the atoms O29 and O30 showed more electronegativity compare to other electronegative atoms which is -0.93, and C1, C19 and C21 has nearly same negative charges which is -0.17. The atoms H35 and H36 has nearest same positive charges, S26 has highest positive charge due to highly influence by the electronegative atoms. In general more electropositive atom accepts on electron and more electronegative atom donates on electron.

**Table 4:** Natural population analysis of (E)-4-((2-hydroxy-3,5-diiodobenzylidene) amino)-N-(pyrimidine)-2-yl) benzene sulfonamide (DIDA).

Atom No	Natural	Natural Population			
	Charge	Core	Valance	Rydberg	Total
C 1	-0.17751	1.99886	4.15726	0.02139	6.17751
C 2	-0.13038	1.99888	4.11397	0.01754	6.13038
C 3	0.34866	1.99852	3.62683	0.02599	5.65134
C 4	-0.26033	1.99870	4.23569	0.02594	6.26033
C 5	-0.21988	1.99876	4.19435	0.02677	6.21988
C 6	-0.22440	1.99867	4.19831	0.02742	6.22440
H 7	0.23102	0.00000	0.76607	0.00291	0.76898
H 8	0.24100	0.00000	0.75618	0.00281	0.75900
C 9	0.15666	1.99912	3.81815	0.02606	5.84334
H 10	0.17076	0.00000	0.82375	0.00550	0.82924
O 11	-0.64830	1.99972	6.63940	0.00917	8.64830
H 12	0.48376	0.00000	0.51057	0.00567	0.51624
I 13	0.20154	46.0000	6.79132	0.00714	52.79846
I 14	0.18854	46.0000	6.80457	0.00689	52.81146
N 15	-0.45338	1.99925	5.43621	0.01792	7.45338
C 16	0.17977	1.99889	3.79935	0.02199	5.82023
C 17	-0.19673	1.99900	4.18121	0.01651	6.19673
C 18	-0.22492	1.99901	4.21069	0.01522	6.22492
C 19	-0.17314	1.99898	4.15580	0.01836	6.17314
H 20	0.22637	0.00000	0.77028	0.00335	0.77363
C 21	-0.17051	1.99898	4.15307	0.01847	6.17051
H 22	0.22713	0.00000	0.77017	0.00270	0.77287
C 23	-0.30921	1.99873	4.28436	0.02612	6.30921
H 24	0.23620	0.00000	0.76116	0.00264	0.76380
H 25	0.23767	0.00000	0.75964	0.00269	0.76233
S 26	2.22127	9.99834	3.57824	0.20215	13.77873
N 27	-0.83058	1.99929	5.81439	0.01690	7.83058
H 28	0.43320	0.00000	0.56304	0.00376	0.56680
O 29	-0.93521	1.99981	6.92665	0.00874	8.93521
O 30	-0.93272	1.99981	6.92392	0.00898	8.93272
C 31	0.60739	1.99918	3.35523	0.03820	5.39261
C 32	0.12029	1.99918	3.85569	0.02484	5.87971
C 33	0.12189	1.99918	3.85408	0.02485	5.87811
C 34	-0.30452	1.99912	4.29089	0.01451	6.30452
H 35	0.19977	0.00000	0.79838	0.00184	0.80023
H 36	0.19996	0.00000	0.79817	0.00187	0.80004
H 37	0.22976	0.00000	0.76836	0.00188	0.77024
N 38	-0.53049	1.99934	5.51578	0.01536	7.53049
N 39	-0.54040	1.99934	5.52511	0.01595	7.54040

### 3.9 Natural bond orbital analysis

Inter and intra molecular interaction between the bonds of atoms was investigated from NBO analysis [15,44,45,46]. The donor-acceptor interactions of the titled molecule DIDA were calculated from B3LYP/GENSEP basic set level with help of second-order perturbation theory are shown in table S2 [47]. In this compound the highest stabilization energy is LP (3) O30 to anti-bonding σ∗(S26–N27) with equilibrium energy is 24.74 Kcal/mol, and LP (2) O11 to anti-bonding π∗(C3–C4) and stabilization energy is 20.31 Kcal/mol, and occupancy is 1.8060 and 1.8324 respectively. The interaction between bonding π(C33–C34), π(C32–N38), π(C33–N39), π(C16–C18), π(C5–C6), and π(C1–C2) to antibonding π∗(C32–N38), π∗(C31–N39), π∗(C33–C34), π∗(C21–C23), π∗(C1–C2) and π∗(C9–N15) with stabilization energy is 35.89, 36.79, 29.54, 14.01, 24.75 and 13.98 Kcal/mol, and occupancy is 1.6247, 1.7319, 1.6995, 1.6055, 1.6709 and 1.6425 respectively.

### 3.10 Localized orbital locator (LOL)

**Figure 10:**
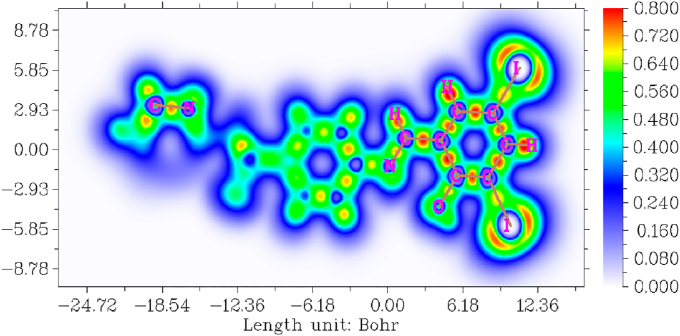
Localized orbital locator map of (E)-4-((2-hydroxy-3,5-diiodobenzylidene) amino)-N-(pyrimidine)-2-yl) benzene sulfonamide.

In synthesized compound DIDA orbital location indicated more important study in Localized Orbital Locator (LOL) Study [15,48]. This is very important study to explain the biological connection between the atoms of the molecule. -24.72–12.36 value ranges in Bohr, with color ranges between 0.000 – 0.800 through blue to red [49]. The localized orbital locater is clearly indicated to Figure 10. The blue color directs intensely delocalized n-orbitals located at oxygen, nitrogen, carbon and surrounding the molecule. The red color directs intensely localized n-orbitals located at iodine and hydrogen atoms [50].

### 3.11 Electron localized function (ELF)

**Figure 11:**
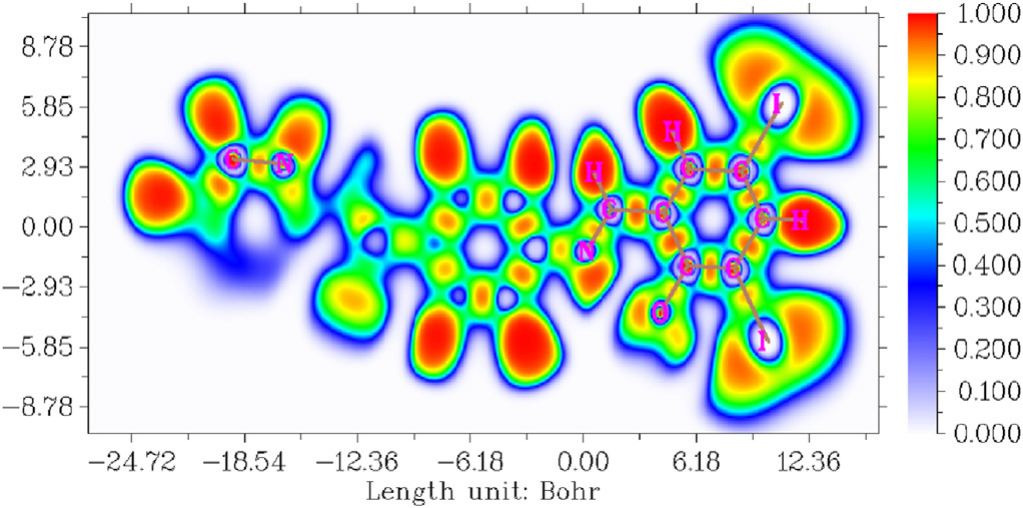
Electron localized function map of (E)-4-((2-hydroxy-3,5-diiodobenzylidene) amino)-N-(pyrimidine)-2-yl) benzene sulfonamide.

The newly synthesized compound DIDA electron localized function is clearly indicated in Figure 11 [15,51]. The ELF higher value clearly indicates strongly localized and electron localized function lower value clearly indicates strongly delocalized function [52]. The ELF color ranges between -24.72–12.36 Bohr, and possibility values are 0.000–1.000 due to the blue to red color [53]. Highly n-localized electron indicated at red color, the red color located at iodine, hydrogen and carbon atoms. The highly n-delocalized electron indicated at blue color, the blue color located at carbon and nitrogen atoms.

### 3.12 Non-covalent interactions (RDG)

**Figure 12:**
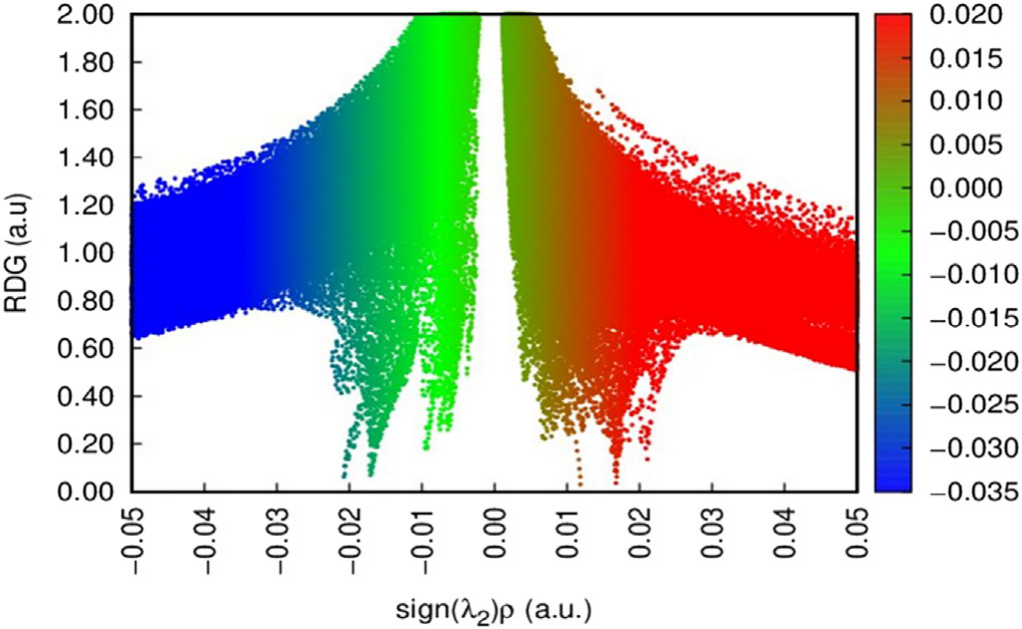
RDG (non-covalent interaction) map of (E)-4-((2-hydroxy-3,5-diiodobenzylidene) amino)-N-(pyrimidine)-2-yl) benzene sulfonamide.

Intra-molecular, inter-molecular and covalent interaction was predict the most important study is non-covalent interaction (RDG) based on the electron density [15,54]. Valuable biological property is predict the non-covalent interaction in the compound DIDA, was directly non-bonded, but bonded some forces like van Der Waals, Hydrogen bonding and steric constant [55]. The RDG of the titled compound DIDA are shown in Figure 12, and graph drawn by reduced density gradient Vs energy [56]. The strongest attraction of hydrogen bond is clearly indicated to blue color. The Vander Waals force and steric constant is clearly indicated at green and red color [57,58]. RDG interactions like hydrophobic interaction, Vander Waals force, dipole-dipole interaction, ion-dipole interaction, pi-stacking and hydrogen bond interaction of the titled compound was docked with DIDA and RNA (protein).

### 3.13 Drug likeness

Predict the biological property is most important technique in ADME [15,59]. The ADME (drug-likeness) stuffs of the titled compound DIDA were considered from Swiss ADME online tools [60]. The titled compound DIDA has good ADME character and bio-activity using PASS online study results [61,62]. The PASS study results most important one is RNA (protein) and the probability active value is 0.954 and probability inactive score is 0.003, these values are listed in Table 5. The physicochemical properties like bioactivity, number rotatable bonds, topological polar surface area, hydrogen bond donor, number heavy atoms, hydrogen bond acceptor etc., are listed in table S3.

**Table 5:** PASS study result of the compound (E)-4-((2-hydroxy-3,5-diiodobenzylidene) amino)-N-(pyrimidine)-2-yl) benzene sulfonamide.

Pa	Pi	Activity
0,954	0,003	Antiinfective
0,684	0,003	Para amino benzoic acid antagonist
0,577	0,007	Antituberculosic
0,558	0,003	Thyroxine 5-deiodinase inhibitor
0,500	0,012	Thiol protease inhibitor
0,474	0,006	Antiprotozoal (Toxoplasma)
0,473	0,006	Growth stimulant
0,472	0,019	Antibacterial
0,457	0,026	Antimycobacterial
0,431	0,004	Falcipain 3 inhibitor

### 3.14 Molecular docking

The biological activity of the titled compound DIDA, docking simulation has been carried out from the Autodock/Vina software [63,64]. Docking simulation predicts the binding affinity (Table 6), favorable non-bond interaction (Table 7), is confirmed in this study [15,65]. The active site of the protein 1ZZ5 was docked to the protein. The crystal structure of the protein 1ZZ5 was obtained from the protein data bank (PDB) [66]. Initially preparation of protein the water molecule removed and non-polar hydrogen bond was added using discovery studio, the active site of protein were definite with 60 A^0^ x 60 A^0^ x 60 A^0^ grid dimension [67].

**Table 6:** Binding affinity of the compound (E)-4-((2-hydroxy-3,5-diiodobenzylidene) amino)-N-(pyrimidine)-2-yl) benzene sulfonamide.

Mode	Binding Affinity	Distance from msd (l,p)	Best mode msd (u,p)
1	-7.4	0	0
2	-7.2	2.288	2.819
3	-7.2	12.168	15.334
4	-7.2	5.198	11.025
5	-7.1	11.922	14.44
6	-7.1	18.185	23.323
7	-7.1	13.426	17.971
8	-7	11.465	12.645
9	-6.9	11.599	12.784

**Table 7:** Favourable non-bond interaction of the compound (E)-4-((2-hydroxy-3,5-diiodobenzylidene) amino)-N-(pyrimidine)-2-yl) benzene sulfonamide.

Distance	Category	Type	From	From-Chem	To	To-Chem
2.84015	H - Bond	Conventional HB	C:C12:H42	H-Donor	:UNK0:N	H-Acceptor
2.15376	H - Bond	Conventional HB	C:C12:H42	H-Donor	:UNK0:O	H-Acceptor
2.70078	H - Bond	Conventional HB	:UNK0:H	H-Donor	C:G15:N7	H-Acceptor
2.66398	H - Bond	Conventional HB	:UNK0:H	H-Donor	D:G24:O6	H-Acceptor
3.52279	H - Bond	Carbon HB	:UNK0:C	H-Donor	A:G2:N7	H-Acceptor
3.23353	H - Bond	Pi-Donor HB	C:C14:H42	H-Donor	:UNK0	Pi-Orbitals
5.38303	Other	Pi-Sulfur	:UNK0:S	Sulfur	C:C14	Pi-Orbitals
5.59132	Hydrophobic	Pi-Pi T-shaped	C:G13	Pi-Orbitals	:UNK0	Pi-Orbitals
4.6898	Hydrophobic	Pi-Pi T-shaped	C:G15	Pi-Orbitals	:UNK0	Pi-Orbitals

Interaction of 1ZZ5 protein shows that 4 conventional H-bond like C:C12:H42 (Cytosine), C:C12:H42 (Cytosine), C:G15:N7 (Guanosine) and D:G24:O6 (Guanosine) with bond distance is 2.8, 2.1, 2.7 and 2.6, one carbon hydrogen bond like A:G2:N7 (Guanosine) with bond distance is 3.5, one Pi-donor H-bond namely C:C14:H42 (Cytosine) with bond distance is 3.2, one Pi-sulfur bond like C:C14 (Cytosine) with bond distance is 5.3, and two Pi-Pi-T-shaped bond namely C:G13 (Guanosine) and C:G15 (Guanosine) with bond distance is 5.5 and 4.6.

**Figure 13:**
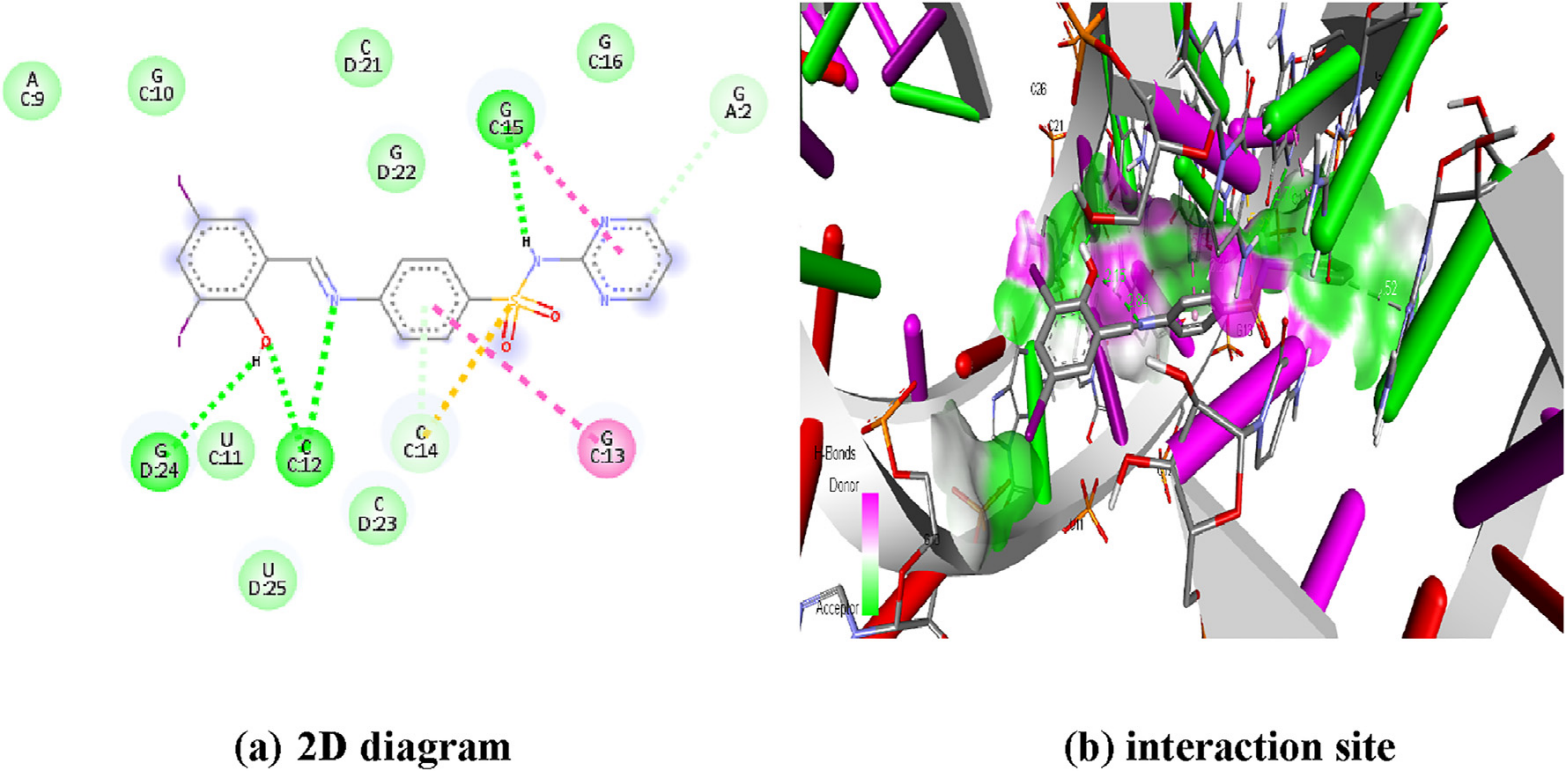
Protein ligand interaction site (a) 2D diagram (b) interaction site of (E)-4-((2-hydroxy-3,5-diiodobenzylidene) amino)-N-(pyrimidine)-2-yl) benzene sulfonamide.

The highest binding affinity observed in this molecule DIDA is -7.4 Kcal/mol, and lowermost binding attraction is -6.9 Kcal/mol. The lively site of the ligand and protein are shown in Figure 13.

## 4 Conclusion

In this research work the compound DIDA synthesized and considered by IR, electronic spectra, and Single crystal (XRD) spectral analysis experimental method and compared with density functional theory with B3LYP/GENSEP basic set level. The FT-IR, UV-Vis, spectral analysis and optimized geometry was compared with theoretical method using with DFT method, the experimental and calculated values are closer. The HOMO-LUMO, NBO, NPO, Mulliken Atomic Charges and MEP also discussed with same DFT method, HOMO-LUMO energy gap is 0.1, so the compound is soft. The wave-function like LOL, ELF and RDG also calculated in this study. Drug-likeness shows good drug-likeness and bio-availability character. The molecular docking studies confirmed with the compound DIDA good biological activity, the compound docked with RNA protein and displayed four conventional hydrogen bonds; and the highest binding affinity is -7.4 Kcal/mol, and lowest binding affinity is -6.9 Kcal/mol.

## Declarations

### Author contribution statement

N. Elangovan: Conceived and designed the experiments; Contributed reagents, materials, analysis tools or data; Wrote the paper.

S. Sowrirajan: Performed the experiments; Contributed reagents, materials, analysis tools or data.

### Funding statement

This research did not receive any specific grant from funding agencies in the public, commercial, or not-for-profit sectors.

### Data availability statement

Data included in article/supplementary material/referenced in article.

### Declaration of interests statement

The authors declare no conflict of interest.

### Additional information

No additional information is available for this paper.
