# Supplementary material for: Synthesis, single crystal (XRD), Hirshfeld surface analysis, computational study (DFT) and molecular docking studies of (E)-4-((2-hydroxy-3,5-diiodobenzylidene)amino)-N-(pyrimidine)-2-yl) benzenesulfonamide
Source: Heliyon. 2021 Aug 6;7(8):e07724. doi: 10.1016/j.heliyon.2021.e07724 (PMC8379672; doi:10.1016/j.heliyon.2021.e07724)
Supplement: Supplementary Material [file mmc1.docx]

**Supplementary Material**

**Table.S1 optimized parameters of the compound (E)-4-((2-hydroxy-3,5-diiodobenzylidene) amino) -N-(pyrimidine) -2-yl) benzenesulfonamide**

| **Bond Length** | **Calculated** | **Experimental** |
| --- | --- | --- |
| (C1,C2) | 1.4023 | 1.391 |
| (C1,C6) | 1.3844 | 1.381 |
| (C1,H7) | 1.0827 | 0.93 |
| (C2,C3) | 1.4183 | 1.403 |
| (C2,C9) | 1.4668 | 1.486 |
| (C3,C4) | 1.4042 | 1.381 |
| (C3,O11) | 1.3403 | 1.366 |
| (C4,C5) | 1.3894 | 1.37 |
| (C4,I13) | 2.1437 | 2.09 |
| (C5,C6) | 1.391 | 1.392 |
| (C5,H8) | 1.0807 | 0.93 |
| (C6,I14) | 2.1337 | 2.091 |
| (C9,H10) | 1.0995 | 0.93 |
| (C9,N15) | 1.2748 | 1.256 |
| (O11,H12) | 0.969 | 0.69 |
| (N15,C16) | 1.399 | 1.421 |
| (C16,C17) | 1.4052 | 1.401 |
| (C16,C18) | 1.4049 | 1.366 |
| (C17,C19) | 1.3858 | 1.402 |
| (C17,H20) | 1.0828 | 0.93 |
| (C18,C21) | 1.3899 | 1.391 |
| (C18,H22) | 1.0835 | 0.93 |
| (C19,C23) | 1.3965 | 1.368 |
| (C19,H24) | 1.0827 | 0.93 |
| (C21,C23) | 1.3924 | 1.391 |
| (C21,H25) | 1.083 | 0.93 |
| (C23,S26) | 1.7926 | 1.757 |
| (S26,N27) | 1.7247 | 1.646 |
| (S26,O29) | 1.4579 | 1.422 |
| (S26,O30) | 1.4524 | 1.409 |
| (N27,H28) | 1.0125 | 0.69 |
| (N27,C31) | 1.3915 | 1.377 |
| (C31,N38) | 1.3393 | 1.327 |
| (C31,N39) | 1.3327 | 1.34 |
| (C32,C34) | 1.3913 | 1.392 |
| (C32,H35) | 1.0865 | 0.93 |
| (C32,N38) | 1.3328 | 1.329 |
| (C33,C34) | 1.3914 | 1.372 |
| (C33,H36) | 1.0863 | 0.93 |
| (C33,N39) | 1.3318 | 1.334 |
| (C34,H37) | 1.0816 | 0.93 |
| **Bond Angle** | **Calculated** | **Experimental** |
| (C2,C1,C6) | 121.4443 | 119.5 |
| (C2,C1,H7) | 118.3853 | 120.3 |
| (C6,C1,H7) | 120.1703 | 120.3 |
| (C1,C2,C3) | 119.1809 | 121 |
| (C1,C2,C9) | 116.2791 | 117.8 |
| (C3,C2,C9) | 124.54 | 121 |
| (C2,C3,C4) | 117.8396 | 117.6 |
| (C2,C3,O11) | 118.7782 | 122.8 |
| (C4,C3,O11) | 123.382 | 119.6 |
| (C3,C4,C5) | 122.4855 | 122.1 |
| (C3,C4,I13) | 119.1007 | 119.4 |
| (C5,C4,I13) | 118.4138 | 118.5 |
| (C4,C5,C6) | 118.9287 | 119.6 |
| (C4,C5,H8) | 120.2056 | 120.2 |
| (C6,C5,H8) | 120.8657 | 120.2 |
| (C1,C6,C5) | 120.1208 | 120 |
| (C1,C6,I14) | 120.1151 | 120.6 |
| (C5,C6,I14) | 119.7641 | 119.3 |
| (C2,C9,H10) | 113.3141 | 119.4 |
| (C2,C9,N15) | 125.8764 | 121.2 |
| (H10,C9,N15) | 120.7985 | 119.4 |
| (C3,O11,H12) | 109.4316 | 115 |
| (C9,N15,C16) | 119.5763 | 119.1 |
| (N15,C16,C17) | 118.0405 | 119.8 |
| (N15,C16,C18) | 122.5775 | 119.5 |
| (C17,C16,C18) | 119.3363 | 120.6 |
| (C16,C17,C19) | 120.5076 | 118.8 |
| (C16,C17,H20) | 118.5251 | 120.6 |
| (C19,C17,H20) | 120.9575 | 120.2 |
| (C16,C18,C21) | 120.3853 | 120.5 |
| (C16,C18,H22) | 119.6632 | 119.8 |
| (C21,C18,H22) | 119.9347 | 119.8 |
| (C17,C19,C23) | 119.1691 | 119.7 |
| (C17,C19,H24) | 120.9261 | 120.1 |
| (C23,C19,H24) | 119.9025 | 120.1 |
| (C18,C21,C23) | 119.2091 | 118.8 |
| (C18,C21,H25) | 121.0252 | 120.6 |
| (C23,C21,H25) | 119.7558 | 120.6 |
| (C19,C23,C21) | 121.3579 | 121.3 |
| (C19,C23,S26) | 119.3919 | 120 |
| (C21,C23,S26) | 119.2444 | 118.6 |
| (C23,S26,N27) | 97.7727 | 106.6 |
| (C23,S26,O29) | 108.4612 | 109.2 |
| (C23,S26,O30) | 109.0229 | 108.2 |
| (N27,S26,O29) | 109.6165 | 102.9 |
| (N27,S26,O30) | 108.2414 | 110.7 |
| (O29,S26,O30) | 121.1898 | 118.6 |
| (S26,N27,H28) | 114.3331 | 117 |
| (S26,N27,C31) | 123.1705 | 120 |
| (H28,N27,C31) | 113.7757 | 116 |
| (N27,C31,N38) | 114.7914 | 117.6 |
| (N27,C31,N39) | 117.8421 | 115.7 |
| (N38,C31,N39) | 127.3565 | 126.6 |
| (C34,C32,H35) | 121.2633 | 118.1 |
| (C34,C32,N38) | 122.6582 | 123.7 |
| (H35,C32,N38) | 116.0772 | 118.1 |
| (C34,C33,H36) | 121.3847 | 118.8 |
| (C34,C33,N39) | 122.6322 | 122.4 |
| (H36,C33,N39) | 115.9831 | 118.8 |
| (C32,C34,C33) | 116.1366 | 115.7 |
| (C32,C34,H37) | 121.9063 | 122.1 |
| (C33,C34,H37) | 121.9551 | 122.1 |
| (C31,N38,C32) | 115.4945 | 115.1 |
| (C31,N39,C33) | 115.7114 | 116.3 |
| **Dihedral Angle** | **Calculated** | **Experimental** |
| (C6,C1,C2,C3) | -0.1565 | 3.1 |
| (C6,C1,C2,C9) | 179.9153 | 177.8 |
| (C2,C1,C6,C5) | -0.011 | -4.1 |
| (C2,C1,C6,I14) | 179.9832 | 178.9 |
| (C1,C2,C3,C4) | 0.2107 | -2.5 |
| (C1,C2,C3,O11) | -179.6377 | -179.1 |
| (C9,C2,C3,C4) | -179.8674 | -176.9 |
| (C9,C2,C3,O11) | 0.2841 | 4.7 |
| (C1,C2,C9,N15) | -176.8209 | -176.5 |
| (C3,C2,C9,N15) | 3.2552 | -1.9 |
| (C2,C3,C4,C5) | -0.1081 | 2.8 |
| (C2,C3,C4,I13) | 179.9055 | -179.4 |
| (O11,C3,C4,C5) | 179.7329 | -178.7 |
| (O11,C3,C4,I13) | -0.2535 | -1 |
| (C3,C4,C5,C6) | -0.0565 | -3.8 |
| (I13,C4,C5,C6) | 179.93 | 178.4 |
| (C4,C5,C6,C1) | 0.1173 | 4.4 |
| (C4,C5,C6,I14) | -179.8768 | -178.5 |
| (C2,C9,N15,C16) | -177.3418 | 172.8 |
| (C9,N15,C16,C17) | -135.0727 | -44 |
| (C9,N15,C16,C18) | 47.4049 | 139.3 |
| (N15,C16,C17,C19) | -179.8564 | 179.1 |
| (C18,C16,C17,C19) | -2.2513 | -4.2 |
| (N15,C16,C18,C21) | 178.7092 | -177.6 |
| (C17,C16,C18,C21) | 1.2177 | 5.8 |
| (C16,C17,C19,C23) | 1.8643 | 0.1 |
| (C16,C18,C21,C23) | 0.1697 | -3.1 |
| (C17,C19,C23,C21) | -0.4489 | 2.5 |
| (C17,C19,C23,S26) | -179.5684 | -174.6 |
| (C18,C21,C23,C19) | -0.5669 | -1 |
| (C18,C21,C23,S26) | 178.5539 | 176.1 |
| (C19,C23,S26,N27) | -83.76 | -108.6 |
| (C19,C23,S26,O29) | 162.4874 | 140.9 |
| (C19,C23,S26,O30) | 28.6502 | 0.5 |
| (C21,C23,S26,N27) | 97.1018 | 74.3 |
| (C21,C23,S26,O29) | -16.6508 | -36.3 |
| (C21,C23,S26,O30) | -150.4881 | -166.6 |
| (C23,S26,N27,C31) | -170.7512 | 65.7 |
| (O29,S26,N27,C31) | -57.9248 | -179.5 |
| (O30,S26,N27,C31) | 76.2094 | -51.8 |
| (S26,N27,C31,N38) | 159.2299 | -17.3 |
| (S26,N27,C31,N39) | -21.8361 | 165.7 |
| (N27,C31,N38,C32) | 177.6491 | 176.3 |
| (N39,C31,N38,C32) | -1.165 | 0.4 |
| (N27,C31,N39,C33) | -178.1312 | 175.1 |
| (N38,C31,N39,C33) | 0.6512 | -1.7 |
| (N38,C32,C34,C33) | 0.1872 | -1.4 |
| (C34,C32,N38,C31) | 0.6784 | 1.2 |
| (N39,C33,C34,C32) | -0.7468 | -0.1 |
| (C34,C33,N39,C31) | 0.3678 | 1.5 |

**Table.S2 Natural bond orbital analysis flock matrix analysis of (E)-4-((2-hydroxy-3,5-diiodobenzylidene) amino) -N-(pyrimidine) -2-yl) benzenesulfonamide**

| **Donar** | **Type** | **ED/e** | **Acceptor** | **Type** | **ED/e** | **E(2)^a^** | **E(j-i)^b^** | **F(i,j)^c^** |
| --- | --- | --- | --- | --- | --- | --- | --- | --- |
| C 1 - C 2 | π | 1.96099 | C 1 - C 6 | σ* | 0.02361 | 2.68 | 1.13 | 0.049 |
|  |  |  | C 1 - H 7 | σ* | 0.01561 | 1.56 | 1.22 | 0.039 |
|  |  |  | C 2 - C 3 | σ* | 0.03815 | 2.61 | 1.13 | 0.049 |
|  |  |  | C 2 - C 9 | σ* | 0.03061 | 2.08 | 1.13 | 0.043 |
|  |  |  | C 3 - O 11 | σ* | 0.02241 | 2.15 | 1.01 | 0.042 |
|  |  |  | C 6 - I 14 | σ* | 0.034 | 2.29 | 0.83 | 0.039 |
|  |  |  | C 9 - N 15 | π* | 0.01172 | 0.78 | 1.35 | 0.029 |
| C 1 - C 2 | π | 1.64257 | C 3 - C 4 | π* | 0.42 | 12.31 | 0.3 | 0.055 |
|  |  |  | C 5 - C 6 | π* | 0.38839 | 10.17 | 0.3 | 0.049 |
|  |  |  | C 9 - N 15 | π* | 0.14693 | 13.98 | 0.29 | 0.059 |
| C 1 - C 6 | σ | 1.97982 | C 1 - C 2 | π* | 0.02181 | 2.37 | 1.26 | 0.049 |
|  |  |  | C 5 - C 6 | π* | 0.02908 | 2.13 | 1.25 | 0.046 |
|  |  |  | C 5 - H 8 | σ* | 0.01676 | 3.1 | 1.11 | 0.052 |
| C 1 - H 7 | σ | 1.97558 | C 1 - C 2 | π* | 0.02181 | 1.74 | 1.15 | 0.04 |
| C 2 - C 3 | σ | 1.95898 | C 1 - C 2 | π* | 0.02181 | 2.57 | 1.25 | 0.051 |
|  |  |  | C 4 - I 13 | σ* | 0.03503 | 5.28 | 0.71 | 0.055 |
|  |  |  | C 9 - H 10 | σ* | 0.03696 | 1.14 | 1.08 | 0.031 |
|  |  |  | O 11 - H 12 | σ* | 0.02506 | 0.88 | 1.07 | 0.028 |
| C 2 - C 9 | σ | 1.96832 | C 1 - C 2 | π* | 0.02181 | 2.52 | 1.25 | 0.05 |
|  |  |  | C 9 - N 15 | π* | 0.01172 | 1.83 | 1.23 | 0.043 |
|  |  |  | N 15 - C 16 | σ* | 0.02673 | 5.21 | 1 | 0.064 |
| C 3 - C 4 | π | 1.97942 | C 2 - C 3 | σ* | 0.03815 | 3.51 | 1.16 | 0.057 |
|  |  |  | C 5 - H 8 | σ* | 0.01676 | 1 | 1.25 | 0.032 |
| C 3 - C 4 | π | 1.65464 | C 1 - C 2 | π* | 0.38315 | 9.02 | 0.33 | 0.049 |
|  |  |  | C 5 - C 6 | π* | 0.38839 | 12.05 | 0.32 | 0.056 |
| C 3 - O 11 | σ | 1.99316 | C 1 - C 2 | π* | 0.02181 | 0.86 | 1.46 | 0.032 |
| C 4 - C 5 | σ | 1.97016 | C 3 - C 4 | π* | 0.03997 | 2.35 | 1.24 | 0.048 |
|  |  |  | C 6 - I 14 | σ* | 0.034 | 4.92 | 0.72 | 0.053 |
| C 4 - I 13 | σ | 1.9713 | C 2 - C 3 | σ* | 0.03815 | 6.23 | 0.9 | 0.067 |
| C 5 - C 6 | π | 1.97072 | C 1 - C 6 | σ* | 0.02361 | 1.76 | 1.15 | 0.04 |
|  |  |  | C 3 - C 4 | π* | 0.03997 | 8.46 | 0.31 | 0.047 |
| C 5 - C 6 | π | 1.67096 | C 1 - C 2 | π* | 0.38315 | 24.25 | 0.29 | 0.076 |
|  |  |  | C 3 - C 4 | π* | 0.42 | 15.51 | 0.28 | 0.06 |
| C 5 - H 8 | σ | 1.97456 | C 1 - C 6 | σ* | 0.02361 | 6.14 | 0.91 | 0.067 |
| C 6 - I 14 | σ | 1.97233 | C 1 - C 2 | π* | 0.02181 | 2.31 | 1.13 | 0.046 |
|  |  |  | C 5 - H 8 | σ* | 0.01676 | 1.09 | 0.98 | 0.029 |
| C 9 - H 10 | σ | 1.98495 | C 2 - C 3 | σ* | 0.03815 | 3.2 | 0.94 | 0.049 |
|  |  |  | C 9 - N 15 | π* | 0.01172 | 0.55 | 1.15 | 0.022 |
| C 9 - N 15 | π | 1.98613 | C 1 - C 2 | π* | 0.02181 | 1.21 | 1.5 | 0.038 |
|  |  |  | N 15 - C 16 | σ* | 0.02673 | 0.88 | 1.24 | 0.03 |
|  |  |  | C 16 - C 17 | σ* | 0.02641 | 2.04 | 1.27 | 0.046 |
| C 9 - N 15 | π | 1.90905 | C 1 - C 2 | π* | 0.38315 | 7.55 | 0.35 | 0.048 |
|  |  |  | C 16 - C 18 | π* | 0.36533 | 11.5 | 0.36 | 0.059 |
| O 11 - H 12 | σ | 1.98668 | C 2 - C 3 | σ* | 0.03815 | 3.59 | 1.12 | 0.057 |
|  |  |  | C 3 - C 4 | π* | 0.03997 | 0.93 | 0.76 | 0.025 |
| N 15 - C 16 | σ | 1.97772 | C 2 - C 9 | σ* | 0.03061 | 3.79 | 1.1 | 0.058 |
|  |  |  | C 17 - C 19 | π* | 0.01532 | 0.7 | 1.34 | 0.028 |
|  |  |  | C 18 - C 21 | σ* | 0.01597 | 2.9 | 1.11 | 0.051 |
| C 16 - C 17 | σ | 1.97134 | C 9 - N 15 | π* | 0.01172 | 1.98 | 1.22 | 0.044 |
|  |  |  | C 18 - H 22 | σ* | 0.01341 | 2.87 | 1.1 | 0.05 |
|  |  |  | C 19 - H 24 | σ* | 0.01454 | 2.73 | 1.1 | 0.049 |
| C 16 - C 18 | π | 1.97378 | N 15 - C 16 | σ* | 0.02673 | 0.87 | 1.11 | 0.028 |
|  |  |  | C 18 - H 22 | σ* | 0.01341 | 1.69 | 1.22 | 0.04 |
|  |  |  | C 21 - H 25 | σ* | 0.01488 | 1.08 | 1.21 | 0.032 |
| C 16 - C 18 | π | 1.60551 | C 9 - N 15 | π* | 0.14693 | 7.27 | 0.29 | 0.043 |
|  |  |  | C 17 - C 19 | π* | 0.27997 | 9.61 | 0.31 | 0.05 |
|  |  |  | C 21 - C 23 | π* | 0.40954 | 14.01 | 0.29 | 0.057 |
| C 17 - C 19 | π | 1.97165 | N 15 - C 16 | σ* | 0.02673 | 2.41 | 1.11 | 0.046 |
|  |  |  | C 19 - H 24 | σ* | 0.01454 | 1.45 | 1.22 | 0.038 |
|  |  |  | C 23 - S 26 | σ* | 0.17882 | 2.72 | 0.89 | 0.046 |
| C 17 - C 19 | π | 1.66694 | C 16 - C 18 | π* | 0.36533 | 11.78 | 0.31 | 0.054 |
|  |  |  | C 21 - C 23 | π* | 0.40954 | 10.68 | 0.29 | 0.05 |
| C 17 - H 20 | σ | 1.97664 | C 16 - C 18 | π* | 0.03241 | 2.6 | 1.15 | 0.049 |
|  |  |  | C 19 - H 24 | σ* | 0.01454 | 0.81 | 1.01 | 0.026 |
| C 18 - C 21 | σ | 1.9708 | N 15 - C 16 | σ* | 0.02673 | 4.6 | 0.99 | 0.06 |
|  |  |  | C 23 - S 26 | σ* | 0.17882 | 5.67 | 0.77 | 0.061 |
| C 18 - H 22 | σ | 1.97617 | C 9 - H 10 | σ* | 0.03696 | 0.51 | 0.98 | 0.02 |
|  |  |  | C 21 - C 23 | π* | 0.02586 | 1.98 | 1.14 | 0.042 |
| C 19 - C 23 | σ | 1.97612 | C 17 - C 19 | π* | 0.01532 | 1.67 | 1.27 | 0.041 |
|  |  |  | C 17 - H 20 | σ* | 0.0304 | 2.46 | 1.12 | 0.047 |
|  |  |  | C 21 - C 23 | π* | 0.02586 | 3.64 | 1.25 | 0.06 |
|  |  |  | C 21 - H 25 | σ* | 0.01488 | 3.03 | 1.11 | 0.052 |
| C 19 - H 24 | σ | 1.97637 | C 16 - C 17 | σ* | 0.02641 | 5.47 | 0.92 | 0.064 |
|  |  |  | C 21 - C 23 | π* | 0.02586 | 2.66 | 1.14 | 0.049 |
| C 21 - C 23 | π | 1.97636 | C 18 - C 21 | σ* | 0.01597 | 1.69 | 1.16 | 0.04 |
|  |  |  | C 19 - H 24 | σ* | 0.01454 | 1.12 | 1.25 | 0.033 |
|  |  |  | C 21 - H 25 | σ* | 0.01488 | 1.76 | 1.24 | 0.042 |
| C 21 - C 23 | π | 1.7058 | C 16 - C 18 | π* | 0.36533 | 7.88 | 0.33 | 0.046 |
|  |  |  | C 17 - C 19 | π* | 0.27997 | 10.67 | 0.33 | 0.053 |
|  |  |  | S 26 - N 27 | π* | 0.26277 | 3.45 | 0.39 | 0.033 |
|  |  |  | S 26 - O 29 | π* | 0.14406 | 3.12 | 0.57 | 0.038 |
| C 21 - H 25 | σ | 1.97598 | C 16 - C 18 | π* | 0.03241 | 1.88 | 1.16 | 0.042 |
|  |  |  | C 19 - C 23 | σ* | 0.0261 | 6.22 | 0.92 | 0.068 |
| C 23 - S 26 | σ | 1.96844 | C 17 - C 19 | π* | 0.01532 | 1.06 | 1.35 | 0.034 |
|  |  |  | S 26 - N 27 | σ* | 0.26277 | 1.74 | 0.81 | 0.036 |
|  |  |  | S 26 - O 29 | π* | 0.14406 | 2.53 | 1 | 0.046 |
|  |  |  | S 26 - O 30 | π* | 0.13727 | 2.19 | 0.99 | 0.043 |
|  |  |  | N 27 - C 31 | σ* | 0.05088 | 1.23 | 1.02 | 0.032 |
| S 26 - N 27 | σ | 1.97343 | C 19 - C 23 | σ* | 0.061 | 0.72 | 1.14 | 0.026 |
|  |  |  | C 31 - N 38 | σ* | 0.03915 | 3.97 | 0.76 | 0.052 |
| S 26 - O 29 | π | 1.98789 | C 19 - C 23 | σ* | 0.0261 | 0.61 | 1.35 | 0.026 |
| S 26 - O 30 | π | 1.98875 | C 21 - C 23 | π* | 0.02586 | 1 | 1.58 | 0.035 |
|  |  |  | S 26 - O 30 | π* | 0.13727 | 0.63 | 1.24 | 0.026 |
| N 27 - H 28 | σ | 1.98252 | S 26 - O 30 | π* | 0.13727 | 1.65 | 0.95 | 0.036 |
|  |  |  | C 31 - N 38 | σ* | 0.03915 | 1.25 | 0.68 | 0.028 |
|  |  |  | C 31 - N 39 | π* | 0.42718 | 3.65 | 1.01 | 0.055 |
| N 27 - C 31 | σ | 1.98705 | C 23 - S 26 | σ* | 0.17882 | 0.98 | 0.94 | 0.028 |
|  |  |  | C 31 - N 38 | σ* | 0.03915 | 0.87 | 1.36 | 0.031 |
|  |  |  | C 32 - N 38 | σ* | 0.01287 | 3.66 | 1.15 | 0.058 |
|  |  |  | C 33 - N 39 | π* | 0.01361 | 1.3 | 1.39 | 0.038 |
| C 31 - N 38 | σ | 1.98347 | S 26 - N 27 | σ* | 0.26277 | 2.73 | 0.94 | 0.048 |
|  |  |  | C 32 - H 35 | σ* | 0.02345 | 2.12 | 1.29 | 0.047 |
|  |  |  | C 32 - N 38 | σ* | 0.01287 | 0.94 | 1.38 | 0.032 |
| C 31 - N 39 | π | 1.98638 | N 27 - H 28 | σ* | 0.01064 | 1.4 | 1.26 | 0.038 |
|  |  |  | C 33 - N 39 | σ* | 0.01361 | 1.03 | 1.38 | 0.034 |
| C 31 - N 39 | π | 1.69952 | C 31 - N 39 | π* | 0.42718 | 0.8 | 0.31 | 0.014 |
|  |  |  | C 32 - N 38 | π* | 0.36606 | 8.76 | 0.32 | 0.048 |
|  |  |  | C 33 - C 34 | π* | 0.2828 | 29.54 | 0.34 | 0.09 |
| C 32 - C 34 | σ | 1.98548 | C 31 - N 38 | σ* | 0.03915 | 0.68 | 1.33 | 0.027 |
|  |  |  | C 33 - N 39 | π* | 0.01361 | 14.76 | 0.29 | 0.059 |
| C 32 - H 35 | σ | 1.98113 | C 31 - N 38 | σ* | 0.03915 | 3.04 | 1.12 | 0.052 |
|  |  |  | C 34 - H 37 | σ* | 0.01268 | 0.6 | 1.02 | 0.022 |
| C 32 - N 38 | π | 1.98358 | N 27 - C 31 | σ* | 0.05088 | 4.35 | 1.24 | 0.066 |
|  |  |  | C 31 - N 38 | σ* | 0.03915 | 1.12 | 1.35 | 0.035 |
|  |  |  | C 32 - C 34 | σ* | 0.0246 | 1.64 | 1.39 | 0.043 |
|  |  |  | C 34 - H 37 | σ* | 0.01268 | 1.52 | 1.29 | 0.04 |
| C 32 - N 38 | π | 1.73199 | C 31 - N 39 | π* | 0.42718 | 36.79 | 0.3 | 0.098 |
|  |  |  | C 33 - C 34 | π* | 0.2828 | 9.96 | 0.33 | 0.051 |
| C 33 - C 34 | π | 1.98539 | C 32 - C 34 | σ* | 0.0246 | 2.26 | 1.28 | 0.048 |
|  |  |  | C 34 - H 37 | σ* | 0.01268 | 1.29 | 1.17 | 0.035 |
| C 33 - C 34 | π | 1.62478 | C 31 - N 39 | π* | 0.42718 | 13.02 | 0.25 | 0.052 |
|  |  |  | C 32 - N 38 | π* | 0.36606 | 35.89 | 0.26 | 0.087 |
|  |  |  | C 33 - C 34 | π* | 0.2828 | 1.76 | 0.28 | 0.02 |
| C 33 - H 36 | σ | 1.98127 | C 31 - N 39 | π* | 0.03874 | 7.52 | 0.88 | 0.073 |
| C 33 - N 39 | σ | 1.98421 | N 27 - C 31 | σ* | 0.05088 | 2.28 | 1.19 | 0.047 |
|  |  |  | C 31 - N 38 | σ* | 0.03915 | 16.47 | 0.32 | 0.066 |
|  |  |  | C 32 - C 34 | σ* | 0.0246 | 7.09 | 0.35 | 0.045 |
| C 34 - H 37 | σ | 1.97819 | C 32 - C 34 | σ* | 0.0246 | 1.25 | 1.15 | 0.034 |
|  |  |  | C 32 - N 38 | π* | 0.01287 | 5.59 | 0.89 | 0.063 |
| LP(1) | O 11 | 1.97694 | C 3 - C 4 | π* | 0.03997 | 4.69 | 1.18 | 0.067 |
| LP(2) | O 11 | 1.83241 | C 3 - C 4 | π* | 0.03997 | 20.31 | 0.34 | 0.078 |
| LP(1) | I 13 | 1.99547 | C 3 - C 4 | π* | 0.03997 | 0.87 | 1.2 | 0.029 |
| LP(2) | I 13 | 1.95748 | C 4 - C 5 | σ* | 0.02567 | 2.83 | 0.65 | 0.038 |
| LP(3) | I 13 | 1.95313 | C 3 - C 4 | π* | 0.03997 | 7.64 | 0.28 | 0.044 |
| LP(1) | I 14 | 1.99526 | C 5 - C 6 | π* | 0.02908 | 0.85 | 1.2 | 0.029 |
| LP(2) | I 14 | 1.97828 | C 1 - C 6 | σ* | 0.02361 | 3.01 | 0.64 | 0.039 |
| LP(2) | I 14 |  | C 5 - C 6 | π* | 0.02908 | 2.56 | 0.87 | 0.042 |
| LP(3) | I 14 | 1.95443 | C 5 - C 6 | π* | 0.02908 | 8.25 | 0.28 | 0.045 |
| LP(1) | N 15 | 1.8001 | C 9 - H 10 | σ* | 0.03696 | 9.55 | 0.79 | 0.079 |
| LP(1) | N 15 |  | C 16 - C 18 | π* | 0.03241 | 6.59 | 0.97 | 0.072 |
| LP(1) | N 27 | 1.7993 | S 26 - O 29 | π* | 0.14406 | 6.97 | 0.64 | 0.06 |
| LP(1) | N 27 |  | C 31 - N 38 | σ* | 0.03915 | 6.36 | 0.94 | 0.07 |
| LP(1) | O 29 | 1.98366 | C 23 - S 26 | σ* | 0.17882 | 1.16 | 0.97 | 0.031 |
| LP(2) | O 29 | 1.81606 | C 23 - S 26 | σ* | 0.17882 | 15.39 | 0.44 | 0.074 |
| LP(2) | O 29 |  | S 26 - N 27 | σ* | 0.26277 | 11.32 | 0.38 | 0.06 |
| LP(3) | O 29 | 1.80208 | S 26 - N 27 | σ* | 0.26277 | 14.37 | 0.39 | 0.067 |
| LP(3) | O 29 |  | S 26 - O 30 | π* | 0.13727 | 17.41 | 0.56 | 0.09 |
| LP(1) | O 30 | 1.98401 | C 23 - S 26 | σ* | 0.17882 | 1.03 | 0.97 | 0.029 |
| LP(2) | O 30 | 1.81335 | C 23 - S 26 | σ* | 0.17882 | 13.03 | 0.45 | 0.068 |
| LP(2) | O 30 |  | S 26 - O 29 | π* | 0.14406 | 14.77 | 0.57 | 0.082 |
| LP(3) | O 30 | 1.80602 | S 26 - N 27 | σ* | 0.26277 | 24.74 | 0.39 | 0.089 |
| LP(3) | O 30 |  | S 26 - O 29 | π* | 0.14406 | 5.71 | 0.57 | 0.051 |
| LP(1) | N 38 | 1.91332 | C 31 - N 39 | π* | 0.03874 | 14.16 | 0.69 | 0.088 |
| LP(1) | N 38 |  | C 32 - C 34 | σ* | 0.0246 | 5.19 | 0.97 | 0.065 |
| LP(1) | N 39 | 1.91269 | C 33 - C 34 | π* | 0.02429 | 11.59 | 0.73 | 0.083 |
| LP(1) | N 39 |  | C 33 - H 36 | σ* | 0.02343 | 4.51 | 0.81 | 0.055 |

**Table.S3 Physicochemical properties of (E)-4-((2-hydroxy-3,5-diiodobenzylidene) amino) -N-(pyrimidine) -2-yl) benzenesulfonamide**

| **Property** | **Value** |
| --- | --- |
| **Physicochemical property** |  |
| Molecular mass | 606.18 |
| Molecular formula | C17H12I2N4O3S |
| Number of heavy atoms | 27 |
| Number of aromatic heavy atoms | 18 |
| Number of rotatable bonds | 5 |
| **Drug-likeness property** |  |
| Number of H-bond acceptors | 6 |
| Number of H-bond donors | 2 |
| Molar Refractivity | 120.3 |
| Bioactivity | 0.55 |
| Topological surface Area (TPSA) | 112.92 |
| logP_ow_ | 4.83 |
| **Pharmacokinetics** |  |
| Gl absortion | High |
| BBB permeant | No |
| P-gp substrate | No |
| CYP1A2 inhibitor | No |
| CYP2C19 inhibitor | No |
| CYP2C9 inhibitor | Yes |
| CYP2D6 inhibitor | No |
| CYP3A4 inhibitor | Yes |
| Log K_p_ (skin permeation) | -7.65 cm/s |

**Table.S4 Experimental and calculated vibrational frequencies (cm-1) of the compound (E)-4-((2-hydroxy-3,5-diiodobenzylidene) amino)-N-(pyrimidine)-2-yl) benzene sulfonamide (DIDA)**

| **DFT/B3LYP/GENSEP** | | | | | **Observed** |  |
| --- | --- | --- | --- | --- | --- | --- |
| **Mode** | **Unscaled** | **Scaled** | **IR_I_** | **R_A_** | **IR** | **Assignment (% PED)** |
| 111 | 3633.59 | 3506 | 71.4417 | 146.2505 | 3497 | νNH(100) |
| 110 | 3594.61 | 3469 | 83.6383 | 193.073 | 3433 | νOH(100) |
| 109 | 3260.36 | 3146 | 18.1342 | 276.5938 | - | νCH(85) |
| 108 | 3246.2 | 3132 | 3.8594 | 108.143 | - | νCH(99) |
| 107 | 3246.03 | 3132 | 0.74 | 45.6264 | - | νCH(99) |
| 106 | 3233.75 | 3120 | 4.4166 | 56.4161 | - | νCH(93) |
| 105 | 3224.79 | 3112 | 1.3928 | 36.786 | - | νCH(75) |
| 104 | 3220.47 | 3108 | 14.5601 | 103.1464 | - | νCH(80) |
| 103 | 3216.66 | 3104 | 19.2258 | 105.852 | - | νCH(75) |
| 102 | 3215.2 | 3102 | 2.1542 | 25.2377 | - | νCH(99) |
| 101 | 3213.7 | 3101 | 4.7225 | 26.4106 | 3084 | νCH(94) |
| 100 | 3014.03 | 2908 | 40.1468 | 56.7361 | 2936 | νCH(100) |
| 99 | 1676.88 | 1618 | 268.382 | 4250.079 | 1610 | νNC(65)+βHCN(11) |
| 98 | 1631.79 | 1574 | 140.423 | 1874.804 | 1582 | νCC(58)+ βHCC(16) |
| 97 | 1612.38 | 1556 | 13.9287 | 1353.403 | - | νCC(46)+ βHCC(10) |
| 96 | 1610.53 | 1554 | 29.3652 | 1089.663 | - | νCC(55) |
| 95 | 1600.28 | 1544 | 435 | 154.535 | 1538 | νNC(49)+ βCNC(11) |
| 94 | 1589.35 | 1533 | 99.3058 | 1774.846 | - | vCC(36)+ βCCC(11) |
| 93 | 1570.21 | 1515 | 75.7411 | 9.8376 | 1489 | νCC(21)+ βNC(29)+ βCCN(11) |
| 92 | 1514.56 | 1461 | 54.7248 | 201.9608 | - | νCC(12)+ βHCC(46)+ βCCC(11) |
| 91 | 1481.58 | 1429 | 1229.49 | 84.3122 | - | νNC(43)+ βHCN(11)+ βNCN(16) |
| 90 | 1477.63 | 1426 | 76.7018 | 266.3084 | 1417 | νCC(13)+ βHCC(17)+ βHCN(15) |
| 89 | 1455.45 | 1404 | 36.273 | 0.9348 | 1411 | βHCN(11)+ βHCC(30) |
| 88 | 1451.87 | 1401 | 11.7422 | 15.8081 | - | βHCN(42) |
| 87 | 1435.58 | 1385 | 22.181 | 6.223 | - | νCC(44)+ βHCC(36)+ βHCN(25) |
| 86 | 1402.51 | 1353 | 9.7278 | 36.8516 | - | νNC(18)+ βHNC(40) |
| 85 | 1401.47 | 1352 | 36.3429 | 3.4641 | - | νCC(38)+ βHCC(26) |
| 84 | 1368.5 | 1320 | 2.1225 | 13.7855 | 1338 | νCC(61) |
| 83 | 1361.76 | 1314 | 42.6679 | 21.2516 | - | νCC(58)+ βHOC(15)+ βHCC(12) |
| 82 | 1347.97 | 1300 | 6.4196 | 1.151 | - | βHNC(37) |
| 81 | 1334.44 | 1287 | 3.068 | 3.6112 | 1281 | βHCC(84) |
| 80 | 1309.85 | 1264 | 14.7221 | 49.0855 | - | νCC(35)+ βHOC(18) |
| 79 | 1280.87 | 1236 | 15.0683 | 2.0165 | - | νCC(14)+ βHCC(19) |
| 78 | 1271.43 | 1227 | 20.1415 | 7.1203 | - | νNC(68) |
| 77 | 1259.46 | 1215 | 56.2938 | 56.6655 | 1201 | νCC(28)+ βHOC(13)+ βHCC(15) |
| 76 | 1228.06 | 1185 | 4.0343 | 11.9871 | 1181 | νNC(46)+ βHNC(16)+ βHCC(14) |
| 75 | 1217.39 | 1174 | 6.3947 | 67.6698 | - | νCC(10)+ βHCC(55) |
| 74 | 1194.48 | 1152 | 150.483 | 1418.873 | 1147 | νNC(19)+ βHCC(15) |
| 73 | 1145.77 | 1105 | 4.3848 | 1.7637 | - | νCC(23)+ βHCC(54) |
| 72 | 1134.76 | 1095 | 104.863 | 173.7807 | - | νCC(26)+ βHCC(12) |
| 71 | 1124.41 | 1085 | 141.377 | 16.3674 | - | νCC(15)+ βHOC(22)+ βHCC(10) |
| 70 | 1111.68 | 1072 | 11.4789 | 7.0691 | - | νCC(23)+ βHCC(41) |
| 69 | 1082.2 | 1044 | 69.9531 | 81.3573 | - | νCC(15)+ βHCC(11) |
| 68 | 1081.51 | 1043 | 2.5803 | 58.714 | 1009 | νCC(64)+ βCNC(46) |
| 67 | 1029.43 | 993 | 26.7248 | 86.4231 | - | τHCNC(60) |
| 66 | 1025.88 | 990 | 1.3297 | 0.3077 | - | βCCC(15)+ τHCCC(62) |
| 65 | 1022.74 | 987 | 0.2609 | 4.4706 | - | τHCNC(73)+ τNNNC(11) |
| 64 | 1018.24 | 982 | 7.8084 | 249.3116 | - | βCCC(36)+ τHCNC(21) |
| 63 | 1008.28 | 973 | 0.0243 | 17.6409 | 974 | τHCCC(79) |
| 62 | 1000.88 | 965 | 0.0026 | 0.1306 | - | τHCNC(81)+ τCNCN(15) |
| 61 | 980.25 | 946 | 45.5245 | 50.244 | 947 | νCC(17)+ νNC(10)+ βCCN(47) |
| 60 | 954.37 | 921 | 76.717 | 4.9068 | - | νSO(94) |
| 59 | 941.77 | 908 | 2.3383 | 29.3767 | - | τHCCC(62) |
| 58 | 921.05 | 888 | 24.3385 | 29.842 | - | τHCCC(62)+ τCCCC(12) |
| 57 | 916.22 | 884 | 4.9643 | 246.383 | - | βCCC(22)+ βCCC(14) |
| 56 | 897.78 | 866 | 92.6713 | 5.2042 | - | νNC(66) |
| 55 | 887.82 | 856 | 21.3461 | 30.2556 | - | τHCCC(71) |
| 54 | 874.14 | 843 | 8.0151 | 15.8458 | 844 | τHCCC(77) |
| 53 | 841.45 | 812 | 66.63 | 6.9817 | - | νCC(11)+ νNC(13)+ νSO(18)+ βCCC(24) |
| 52 | 837.13 | 807 | 65.137 | 0.4516 | - | τHCNC(77)+ τCNCC(13) |
| 51 | 825.33 | 796 | 92.5949 | 16.2273 | 795 | νSO(51)+ βCCC(10) |
| 50 | 823.48 | 794 | 1.9121 | 0.0577 | - | τHCNC(18)+ τCNCC(48)+ τNNNC(19) |
| 49 | 802.7 | 774 | 15.4385 | 15.1191 | - | νCC(18)+ βCCC(24)+ βCCC(17) |
| 48 | 775.72 | 748 | 15.3395 | 6.4769 | 743 | τCCCC(44)+ τCCSC(10) |
| 47 | 759.99 | 733 | 15.9074 | 3.6337 | - | τCCSC(52) |
| 46 | 712.71 | 687 | 48.5633 | 90.3013 | - | νSO(15)+ νSN(30)+ βNCN(17)+ βSNC(11) |
| 45 | 705.03 | 680 | 6.5162 | 36.9403 | 660 | βCCC(15)+ βCCC(26) |
| 44 | 650.28 | 627 | 78.37 | 6.8875 | 627 | νCC(18)+ βCCC(27) |
| 43 | 646.91 | 624 | 68.0315 | 20.8012 | - | βNCN(22) |
| 42 | 640.25 | 617 | 5.8635 | 9.4649 | - | βCCC(55) |
| 41 | 633.72 | 611 | 2.2502 | 14.3244 | - | βCNC(65) |
| 40 | 596.86 | 576 | 101.544 | 3.2446 | 583 | νSC(16)+ τCCC(34) |
| 39 | 582.41 | 562 | 3.4036 | 24.2366 | 560 | τHCCC(13)+ τCCCC(25)+ τNCCC(13) |
| 38 | 570.78 | 550 | 41.8229 | 29.9916 | 543 | τCCCC(12)+ τNCCC(40) |
| 37 | 548.56 | 529 | 3.1486 | 6.0033 | - | βCCO(10)+ τCCCC(41) |
| 36 | 545.6 | 526 | 0.3422 | 0.2245 | 522 | τHCNC(12)+ τCNCC(18)+ τNNNC(57) |
| 35 | 517.38 | 499 | 43.0245 | 5.4069 | 493 | βCCO(17)+ τCCCC(10)+ τNCCC(14) |
| 34 | 472.66 | 456 | 190.607 | 14.2215 | 459 | τONOS(23) |
| 33 | 461.64 | 445 | 90.9754 | 1.276 | - | τHNCN(89) |
| 32 | 445.44 | 429 | 22.0067 | 5.9239 | - | βCCO(31)+ τHOCC(19) |
| 31 | 437.56 | 422 | 117.168 | 6.0258 | 427 | τHOCC(73) |
| 30 | 427.14 | 412 | 5.9394 | 20.3903 | - | τCCCS(63) |
| 29 | 423.63 | 408 | 2.735 | 2.3307 | - | τHCNC(15)+ τCNCN(72) |
| 28 | 417.24 | 402 | 28.0545 | 3.003 | - | νSC(12)+ βOSO(29)+ τCCSC(12) |
| 27 | 382.45 | 369 | 14.8023 | 5.0495 | - | βCCS(10)+ τONCS(41) |
| 26 | 369.16 | 356 | 27.8843 | 37.3985 | - | τCCSC(16)+ τCCCC(14) |
| 25 | 337.75 | 325 | 14.9998 | 5.2937 | - | βCCC(10)+ βOSO(18)+ βCCI(13) |
| 24 | 332.62 | 321 | 0.7783 | 13.3629 | - | CCSC(12)+ τCCCC(31) |
| 23 | 287.92 | 277 | 3.1927 | 2.2205 | - | νIC(13)+ βCCI(19) |
| 22 | 282.6 | 272 | 6.0394 | 7.8155 | - | βCCS(35)+ τONOS(16) |
| 21 | 268 | 258 | 1.3475 | 21.9587 | - | νSN(10) |
| 20 | 257.93 | 248 | 13.0399 | 15.8484 | - | βOSO(15)+ τCCCN(12)+ τCCCC(12) |
| 19 | 251.12 | 242 | 8.6218 | 9.8836 | - | νSN(11)+ βCCS(10)+ τONOS(13) |
| 18 | 227.74 | 219 | 4.0581 | 8.1013 | - | νSC(10)+ νIC(17) |
| 17 | 193.74 | 186 | 10.5741 | 15.5138 | - | νIC(14)+ βCSN(13)+ βCCI(13)+ τCCNC(17) |
| 16 | 188.21 | 181 | 3.2476 | 0.7105 | - | τCNCN(60) |
| 15 | 167.98 | 162 | 4.8372 | 11.8673 | - | νIC(13)+ βCCS(16)+ τONCS(12) |
| 14 | 150.08 | 144 | 8.7719 | 14.0523 | - | νIC(11)+ βCCS(14)+ τCCCN(11) |
| 13 | 136.75 | 131 | 1.9906 | 13.9645 | - | τCCCN(48)+ τCCCN(13) |
| 12 | 106.82 | 103 | 2.162 | 11.3121 | - | βNCN(12)+ βCCI(17)+ βSNC(20) |
| 11 | 102.63 | 99 | 1.0896 | 4.8261 | - | τCCCC(49) |
| 10 | 93.64 | 90 | 2.0315 | 2.0579 | - | βSNC(12)+ τCCCC(18)+ τCCNC(10) |
| 9 | 77.91 | 75 | 0.5744 | 7.9496 | - | τCCCN(13)+ τCCCN(32) |
| 8 | 71.62 | 69 | 0.2734 | 4.5023 | - | βCCI(47) |
| 7 | 59.48 | 57 | 0.1124 | 1.3436 | - | τCCSN(87) |
| 6 | 51.09 | 49 | 0.0365 | 7.8157 | - | τCCSN(76) |
| 5 | 39.69 | 38 | 0.389 | 2.577 | - | βCSN(22)+ τCCNC(16)+ τCCCC(10) |
| 4 | 35.29 | 34 | 0.7799 | 6.9846 | - | τCCCN(68) |
| 3 | 22.2 | 21 | 0.3065 | 2.4669 | - | βCNC(15)+ βCCO(10)+ τCCNC(10)+ τCCCC(26) |
| 2 | 13.1 | 12 | 0.4194 | 0.8265 | - | βCNC(11)+ τCCNC(48) |
| 1 | 8.43 | 8 | 0.8995 | 1.3314 | - | τCCCN(78) |
